# Tumor-infiltrating mast cells are associated with resistance to anti-PD-1 therapy

**DOI:** 10.1038/s41467-020-20600-7

**Published:** 2021-01-12

**Authors:** Rajasekharan Somasundaram, Thomas Connelly, Robin Choi, Hyeree Choi, Anastasia Samarkina, Ling Li, Elizabeth Gregorio, Yeqing Chen, Rohit Thakur, Mohamed Abdel-Mohsen, Marilda Beqiri, Meaghan Kiernan, Michela Perego, Fang Wang, Min Xiao, Patricia Brafford, Xue Yang, Xiaowei Xu, Anthony Secreto, Gwenn Danet-Desnoyers, Daniel Traum, Klaus H. Kaestner, Alexander C. Huang, Denitsa Hristova, Joshua Wang, Mizuho Fukunaga-Kalabis, Clemens Krepler, Fang Ping-Chen, Xiangyang Zhou, Alexis Gutierrez, Vito W. Rebecca, Prashanthi Vonteddu, Farokh Dotiwala, Shashi Bala, Sonali Majumdar, Harsh Dweep, Jayamanna Wickramasinghe, Andrew V. Kossenkov, Jorge Reyes-Arbujas, Kenisha Santiago, Tran Nguyen, Johannes Griss, Frederick Keeney, James Hayden, Brian J. Gavin, David Weiner, Luis J. Montaner, Qin Liu, Lukas Peiffer, Jürgen Becker, Elizabeth M. Burton, Michael A. Davies, Michael T. Tetzlaff, Kar Muthumani, Jennifer A. Wargo, Dmitry Gabrilovich, Meenhard Herlyn

**Affiliations:** 1 The Wistar Institute, Philadelphia, PA USA; 2 Division of Cancer Epidemiology and Genetics, National Cancer Institute, Bethesda, MD USA; 3 Department of Pathology and Medicine, University of Pennsylvania, Philadelphia, PA USA; 4 Department of Medicine, Stem Cell and Xenograft Core, University of Pennsylvania, Philadelphia, PA USA; 5 Department of Genetics and Institute for Diabetes, Obesity and Metabolism, Perelman School of Medicine, University of Pennsylvania, Philadelphia, PA USA; 6 Division of Immunology, Allergy and Infectious Diseases (DIAID), Department of Dermatology, Medical University of Vienna, Vienna, Austria; 7 University of Duisburg-Essen, Essen, Germany; 8 Department of Surgical Oncology, MD Anderson Cancer Center, Houston, TX USA; 9 Department of Melanoma Medical Oncology, University of California, San Francisco, CA USA; 10 Department of Pathology and Dermatology, University of California, San Francisco, CA USA; 11 AstraZeneca, Gaithersburg, MD USA; 12 Present Address: GeneOne Life Science Inc., Fort Washington, PA USA

**Keywords:** Melanoma, Tumour immunology, Adaptive immunity, Mast cells

## Abstract

Anti-PD-1 therapy is used as a front-line treatment for many cancers, but mechanistic insight into this therapy resistance is still lacking. Here we generate a humanized (Hu)-mouse melanoma model by injecting fetal liver-derived CD34^+^ cells and implanting autologous thymus in immune-deficient NOD-*scid* IL2Rγ^null^ (NSG) mice. Reconstituted Hu-mice are challenged with HLA-matched melanomas and treated with anti-PD-1, which results in restricted tumor growth but not complete regression. Tumor RNA-seq, multiplexed imaging and immunohistology staining show high expression of chemokines, as well as recruitment of FOXP3^+^ Treg and mast cells, in selective tumor regions. Reduced HLA-class I expression and CD8^+^/Granz B^+^ T cells homeostasis are observed in tumor regions where FOXP3^+^ Treg and mast cells co-localize, with such features associated with resistance to anti-PD-1 treatment. Combining anti-PD-1 with sunitinib or imatinib results in the depletion of mast cells and complete regression of tumors. Our results thus implicate mast cell depletion for improving the efficacy of anti-PD-1 therapy.

## Abstract (web summary)

Immune checkpoint therapies (ICT) are promising for treating various cancers, but response rates vary. Here the authors show, in mouse models, that tumor-infiltrating mast cells colocalize with regulatory T cells, coincide with local reduction of MHC-I and CD8 T cells, and is associated with resistance to ICT, which can be reversed by c-kit inhibitor treatment.

## Introduction

Tumor-infiltrating immune and non-immune cells are known to modulate therapy responses in many solid tumors including melanoma^1,2^. Immune non-responsiveness and therapy resistance are two major stumbling blocks in the treatment of tumor patients receiving anti-PD-1 therapy^3–6^. Response to anti-PD-1 therapy is frequently correlated with the degree of pre-existing tumor-infiltrating T cells including CD8^+^ T cells^7,8^. Despite the presence of tumor-reactive CD8^+^ T cells, cancer cells often escape these cytotoxic T cells by several mechanisms that include, (i) downmodulation of HLA-class I expression on tumor cells that is essential for tumor antigen presentation^1,4^, (ii) promoting selective infiltration of immune regulatory cells that can directly downmodulate T-cell responses^9^ or indirectly by downmodulating HLA class I expression on tumor cells^10^ and (iii) by increased expression of immune-checkpoint molecules^9,11,12^. Attempts to block immune-checkpoint molecules such as CTLA4 or PD-1 has met with varied success. Little over 50% of the patients treated with anti-PD-1 antibodies do not show any response, about 10% of the individuals have stable disease, 25% of the individuals showed partial responses and 10–15% of the individuals showed complete responses^1,13,14^. In some patients who showed initial responses to anti-PD-1 therapy, follow-up indicated recurrence of tumors. Resistance mechanisms to immune-based therapies are poorly understood as available mouse tumor models do not replicate the human tumor microenvironment^15,16^. This led us to the development of the Hu-mice model with innate and adaptive immune cells.

In the tumor-bearing Hu-mouse model, we investigate the crosstalk between innate immune cells and tumor cells in anti-PD-1 resistance. Our results suggest that tumor-infiltrating mast cells are associated with anti-PD-1 resistance. Targeted depletion of these cells using sunitinib or imatinib and in combination with anti-PD-1 results in complete regression of tumors. Here, we uncover a critical role of tumor-infiltrating mast cells in anti-PD-1 resistance.

## Results

### Establishment of Hu-mice

**Fig. 1:**
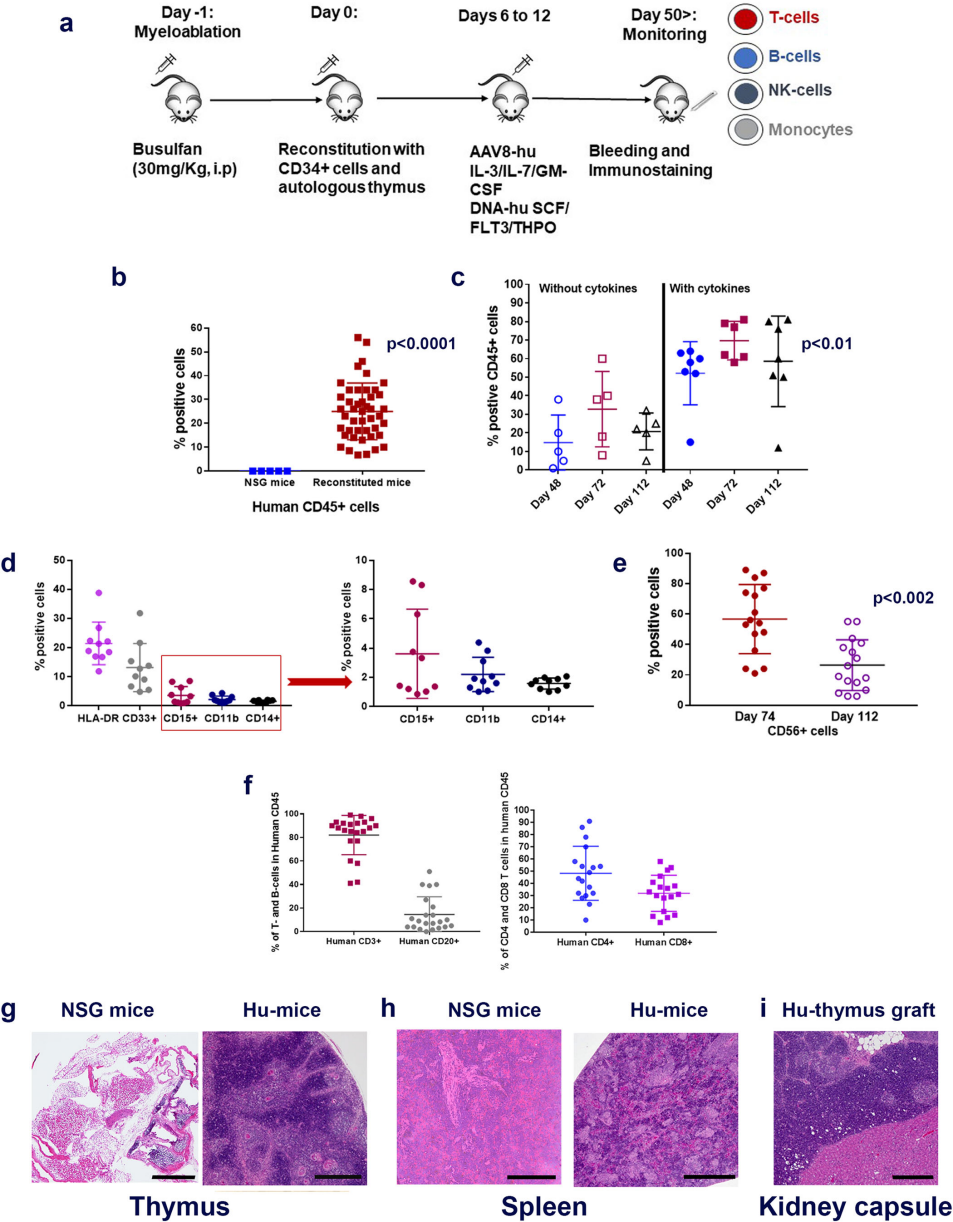
Generation of Hu-mice. **a** Schematic of Hu-mice reconstitution. Six-week-old female NSG mice are all treated with the myelo-depleting drug (busulfan [30 mg/kg]; i.p.) 24 h before they receive purified fetal liver-derived CD34^+^ cells (1 × 10^5^; i.v.) and autologous thymus grafts (~2 mm) under the renal capsule. After day 50, mice are periodically bled (100 μl) and characterized for human immune cells by standard flow cytometry assay using fluorochrome-conjugated anti-mouse or anti-human antibodies. **b** Repopulation of human CD45^+^ cells in circulating blood of reconstituted mice. A representative example of mice (*n* = 45) after 8–12 weeks of human CD34^+^ cell injection showed increased levels of human CD45^+^ cells (brown squares; *p* = 0.00025) in circulating blood when compared to control non-reconstituted female NSG mice (blue squares; *n* = **5**). c Enhanced repopulation of human lymphocytes after AAV8-hu-cytokine transgenes delivery. Significant increase in circulating human CD45^+^ cells (*p* = 0.022 for days 48 and 72 [closed blue circles and brown squares] and *p* = 0.0094 for day 112 [closed black triangles]) in mice (*n* = 7) that received AAV8 hu-cytokines (IL3, IL-7, and GM-CSF; 2 × 10^9^ GC/ml; i.v.; 5 days after CD34 injection; right panel) when compared to mice (*n* = 5) that did not receive hu-cytokines (left panel). **d** Myeloid lineage cells after administration of hu-cytokines. CD33^+^, CD15^+^, CD11b^+^, and CD14^+^ cells are also seen in circulating blood after week 12 of CD34^+^ cells administration and when mice (*n* = 10) receive AAV8 hu-cytokines (see above) plus DNA-hu-cytokines (SCF, FLT-3, THPO; 50 μg; i.m.; multiple sites). An independent batch of mice was used for this experiment. **e** Reconstituted mice show the presence of CD56^+^ NK (innate immune) cells. Mice (*n* = 16) bled at 10 weeks showed increased CD56^+^ cells that decrease significantly to physiological levels by week 16 (*p* = 0.002). An independent batch of mice was used for this experiment. From **d**, all mice received AAV8 or DNA plasmid-encoded human cytokines as described in the method section. **f–i** Repopulation of human T- and B-cells. Generally, by 12–14 weeks, physiological levels of human T-and B-cells (**f**, left panel) and human CD4^+^ and CD8^+^ T cells are observed in circulating blood (**f**, right panel). Each point in the scatter plot represents blood drawn from an individual mouse (*n* = 16). **g**–**i** Repopulation of lymphoid organs with human immune cells. In H&E staining, there is dense repopulation of human lymphocytes in reconstituted mouse thymus (**g**; right panel; scale bar: 200 μm) and spleen (**h**; right panel; scale bar: 250 μm) when compared to non-reconstituted mouse thymus (**g**; left panel; scale bar: 500 μm) and spleen (**h**; left panel; scale bar: 200 μm). Dense repopulation of human lymphocytes in mouse kidney (renal capsule) grafted with human thymus (**I**; scale bar: 200 μm). A representative example of staining is shown in this figure. All observations (**g**–**i**) were consistent, and the experiment was repeated more than 2×. All mice were euthanized by CO_2_ inhalation/cervical dislocation and organs harvested 24 weeks after CD34^+^ cell injections. Data are presented as mean values ± SD for **b**–**f**. A one-sided paired *t*-test was used for statistical analysis when *p*-values are specified. Source data are provided as a Source Data file.

We have used an advanced hu-mouse model which differs significantly from other transgenic Hu-chimeras to delineate the mechanism of immune resistance to anti-PD-1 therapy (Fig. 1a)^17–19^. In contrast to transgenic humanized mouse chimeras that produce growth and differentiation factors continuously, in our model, targeted and sequential delivery of cytokine factors are provided by transgenes encoded in AAV8 or pMV101 DNA-based vectors (Supplementary Fig. 1) to promote human immune cell reconstitution. Unlike other Hu-mice models, our immune reconstituted mice have a stable life span of ~30 weeks (Supplementary Fig. 2) after human CD34^+^ cell injections. The long-term stability of our model offers an opportunity to characterize treatment responses to immune-based therapies after the human tumor challenge. In this model, 8–12 weeks after CD34^+^ cell injection, a robust level of human CD45^+^ lymphocytes are observed in the peripheral blood when compared to circulating blood of non-reconstituted NSG mice (Fig. 1b). Delivery of AAV8 Hu-cytokines (IL-3, IL-7, and GM-CSF) significantly (*p* = 0.00025) increased the levels of human CD45^+^ cells in mouse peripheral blood circulation when compared to the group that did not receive any cytokines (Fig. 1c). The addition of cytokines such as SCF, FLT3, and THPO helps in T-cell and myeloid cell differentiation but does not enhance the level of human CD45^+^ cells. All the subsequent batches of mice described henceforth, received all the above cytokines such as IL-3, IL-7, GM-CSF, SCF, FLT3, and THPO. In independent batches of mice, besides the presence of human CD45^+^ cells, we observed the presence of a small population of monocytes/myeloid lineage cells (HLA DR^+^, CD33^+^, CD15^+^, CD11b^+^, and CD14^+^; (Fig. 1d)), robust levels of NK- cells (CD56^+^) (Fig. 1e), T cells (CD3^+^; CD4^+^, and CD8^+^) and B-cells (CD20^+^) (Fig. 1f). NK-cell (Fig. 1e) and B-cell subpopulations (Supplementary Fig. 3a) are initially high, three to four weeks later their levels drop down as the mouse thymus (Fig. 1g and Supplementary Fig. 3b), mouse spleen (Fig. 1h and Supplementary Fig. 3c) and the renal capsule-grafted Hu-thymus (Fig. 1i) get repopulated with human lymphoid precursor cells that undergo differentiation. Immunodeficient NSG mice, due to their IL2Rγ^null^ genotype, have underdeveloped lymph nodes^20^ and hence we were unable to get enough tissue material for characterization of this lymphoid organ. Efficient antigen presentation to T- and B-cells depends on macrophages (CD68^+^) and we observed them in the spleen and small intestine (Supplementary Fig. 4a). B-cells are fully functional as we detected antigen-specific IgG in circulating blood (see anti-hTERT response below). Human CD4^+^ and CD8^+^ subpopulations of T cells were detected in spleen, thymus, and mesenteric lymph node tissues (Supplementary Fig. 4b). Tissue-resident T cells in the liver, mesenteric lymph nodes, and in the spleen are TCR γ/δ+. These cells are further expanded in vivo in the presence of a bacterial metabolite (hydroxy-2-methyl-2-butenyl 4-pyrophosphate [HMBPP]^21^; Supplementary Fig. 4c). T cells expressing TCR γ/δ+ chains are known to protect against pathogens in mucosal or epithelial layers; as their functional activity is HLA unrestricted, their potential use in adoptive T-cell therapy is being explored in solid tumors^22^. Most T cells in the spleen have a diverse expression of T-cell receptor (TCR) α/β^+^ chains (Supplementary Fig. 5).

### Immune cells in Hu-mice are functional: respond to tumor challenge

**Fig. 2:**
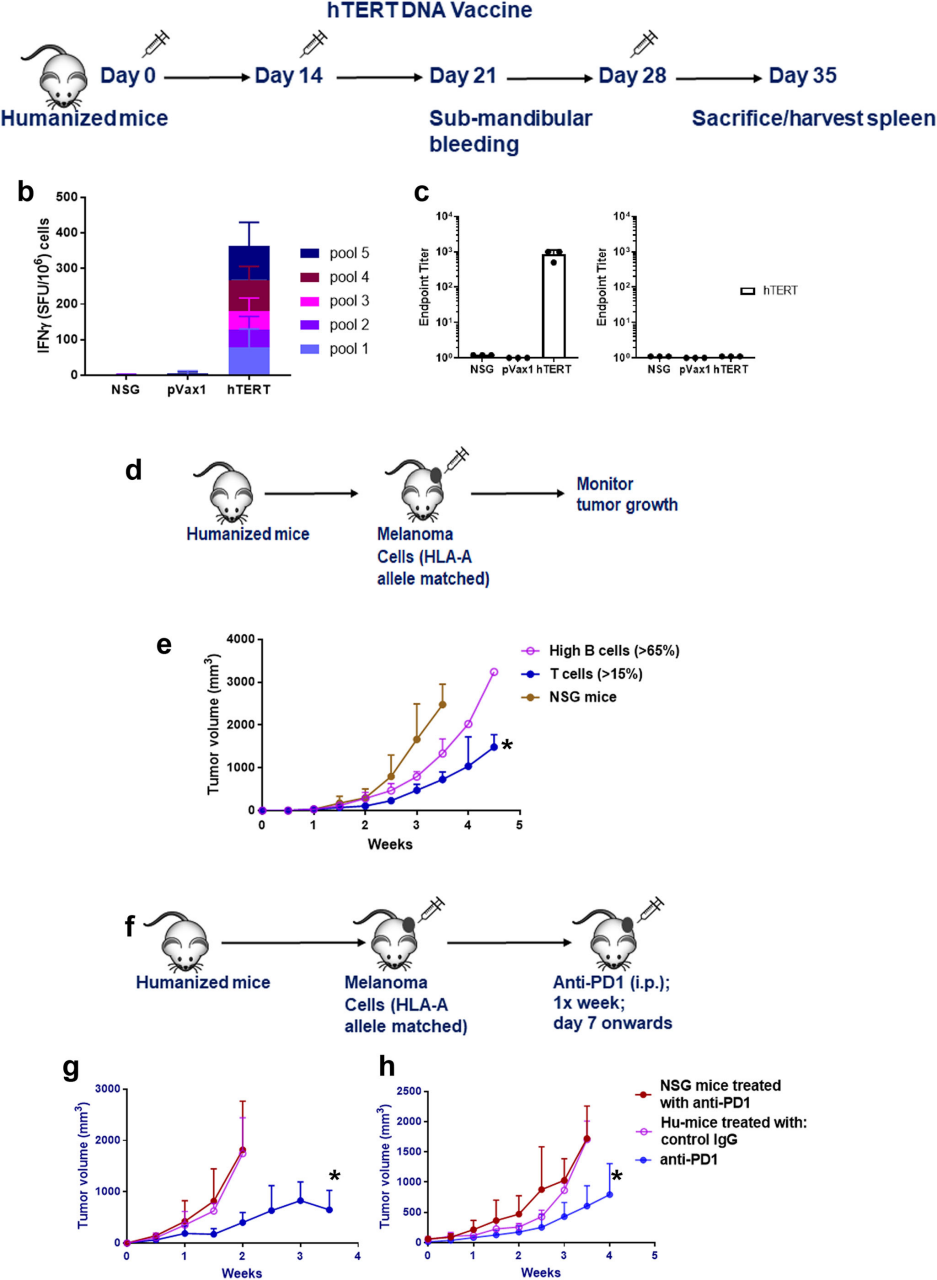
Functional characterization of human immune cells in humanized mice. **a**–**c** T- and B-cell response to hTERT vaccine. **a** Schema for hTERT DNA vaccination. Hu-mice, as described in Fig. 1, received a total of three injections of hTERT vaccine (hTERT DNA [50 μg; i.m] followed by electroporation) every 2 weeks and the mice were euthanized by CO_2_ inhalation/cervical dislocation and organs harvested 1 week after the last injection to determine T- and B-cell responses. **b** Anti-TERT T-cell responses. Spleen cells from Hu-mice (*n* = 3) were stimulated overnight (18 h) with pools of overlapping hTERT peptides (15 mer; 2 μg/ml/peptide) spanning the entire hTERT protein. Human IFNγ was detected in ELISPOT assay using a kit. Data are represented as SFU (spot forming units; mean ± SE) per 10^6^ splenocytes. hTERT-specific T-cell (IFNγ) response from vaccinated mice was compared to age-matched Hu-mice that received pMV101, a modified pVAX1 vector, as control or untreated age, sex-matched NSG mice controls. **c** Anti-TERT antibody (IgG) responses. Endpoint binding titer was determined in sera of hTERT vaccinated mice (*n* = 3) after 3 immunizations and compared to sera from NSG mice as controls. Data are presented as mean values ± SD. **d**–**h** Functional ability of immune T cells to restrict tumor growth. **d** Schema for Hu-mice tumor challenge experiment. **e** Hu-mice with T-cell reconstitution can restrict tumor growth of HLA-A2 matched A375 melanoma cells. Hu-mice, as described in Fig. 1 that have 90 to 120 cells/μl circulating CD45+/CD8+ cells (closed circle, blue line) when challenged with melanoma cells (10^5^; s.c.), can restrict tumor growth significantly (*p = 0.0281) when compared to non-reconstituted NSG mice (*n* = 3; closed circles, brown line) and Hu-mice with high circulating B-cells (*n* = 4; >65% [550 to 600 cells/μl] CD20^+^; open circles, magenta line) have unrestricted tumor growth. Tumor growth measurements are recorded using a digital caliper by an independent researcher. **f**–**h** Treatment with anti-PD-1 antibody can restrict tumor growth of melanoma cells. **f** Schema for anti-PD-1 therapy. Hu-mice with CD45^+^/CD8^+^ (90 to 120 cells/μl) and cells were randomized, and they receive melanoma cells (10^5^; s.c.). When tumors are palpable, mice receive anti-PD-1 (10 mg/kg; i.p. injections) every week for four injections and tumor growth measurements are recorded. **g**, **h** Anti-PD-1 therapy restrict melanoma growth. Hu-mice (*n* = 10) treated with anti-PD-1 antibody can restrict tumor growth of two different melanoma cells [(WM3629 [HLA-A3]; **g**] and [A375 [HLA-A2]; **h**]) significantly (blue line, closed circles; *p = 0.0437 for WM3629 and *p* = 0.0547 for A375) when compared to Hu-mice treated with control IgG (*n* = 5; magenta; open circles) or non-reconstituted age and sex-matched NSG mice (*n* = 10; brown; closed circles) treated with the ant-PD-1 antibody. Two different batches of Hu-mice were used for this experiment and they had comparable CD45^+^/CD8^+^ T-cell counts. Unrestricted tumor growth in presence of anti-PD-1 antibody was observed when Hu-mice were challenged with an aggressive phenotype of the tumor (Supplementary Fig. 8). Data are presented as mean values ± SEM for **e**–**h**. A one-sided paired *t*-test was used for analysis when *p*-values are provided. Source data are provided as a Source Data file.

If mice are fully reconstituted with human lymphoid cells, then one needs to determine the functionality of the humoral (B-) and cellular (T-) immune cell compartment and their ability to respond to an immunizing agent that frequently requires antigen presentation to T- and B-cells. For this, Hu-mice were immunized with hTERT DNA vaccine, a universal tumor-associated antigen^23^ (Fig. 2a), and the lymphoid cells in the spleen were tested for their ability to respond to hTERT antigen after in vitro stimulation followed by IFNγ ELISPOT assay. Anti-TERT-specific T-cell responses were observed on a panel of overlapping peptides spanning the hTERT protein (Fig. 2b). In addition, the sera from hTERT immunized Hu-mice showed the presence of hTERT-specific IgG antibodies confirming the functionality of B-cells (Fig. 2c). There were no T- or B-cell responses to the pVax1 vector alone and the spleen cells from control non-immune reconstituted NSG mice did not respond to hTERT DNA vaccination (Fig. 2b, c).

Next, we determined whether T cells have the ability to restrict tumor growth in the humanized melanoma mouse model (Schema, Fig. 2d). For this, mice with 90–120 absolute CD8^+^ T-cell numbers/μl in peripheral blood were challenged with melanoma cells that are matched to one of the HLA-A allele expressed on the donor CD34^+^ cells. Several of our melanoma cell lines express only one of the HLA-A allele. Also, these cell lines do not show the protein expression of HLA-C molecules as determined by FACS analysis. We have not matched the CD34^+^ cells for HLA-B alleles as it was nearly impossible to find donor CD34 cells that match both HLA-A and B-alleles and there is no humanized model available yet that provides a fully autologous system. Since our model has a thymus graft (autologous to the donor CD34^+^ cells) implanted under the renal capsule to abrogate high-affinity TCR with allo-reactivity and we do not see any adverse events in the fully reconstituted Hu-mice. Despite the limitation of the HLA-A allele matched tumor model, there was a significant restriction of tumor growth when compared to non-reconstituted NSG mice or Hu-mice with high circulating B-cells (>65% CD20^+^; Fig. 2e) and low (<1%) CD8^+^ T cells. TCR sequences obtained from the tumor indicated restricted expression of TCR α/β^+^ chains (Supplementary Fig. 5). T cells from the spleen obtained from three different donor batches (HLA-A1, -A2, and -A3) of Hu-mice, all reacted to CD8 peptides derived from melanoma differentiation antigens confirming reactivity of T cells against melanoma tumors (Supplementary Fig. 6). For further evidence of tumor reactivity, we have used two additional batches of HLA-A2 tumor-bearing Hu-mice and show that CDR3/TCR sequences predict and suggestive of diverse reactivity to melanoma differentiation antigens (MART-1 and gp100) and cancer-testis antigens (MAGE A1 and NY-ESO-1) in the spleen. However, in the tumor, we find CDR3/TCR sequences suggestive of restricted/clonal reactivity to MART-1 (Supplementary Fig. 7). CDR3 sequences obtained from spleen and tumor mapped and suggestive of very limited non-specific reactivity to CMV or EBV related antigens. Next, if T cells are sensitized to tumors and have the ability to restrict tumor growth, we wanted to know whether treatment of tumor-bearing Hu-mice will benefit from anti-PD-1 therapy that is known to sustain tumor-infiltrating CD8^+^ T-cell activity^1,9,12^. In an established tumor model, Hu-mice with similar CD8^+^ T-cell levels (Schema, Fig. 2f), treatment with anti-PD-1 antibody significantly restricted tumor growth of 2 different metastatic melanomas (WM3629 [HLA-A3]; Fig. 2g and A375 [HLA-A2]; Fig. 2h) when compared to tumor growth in Hu-mice treated with control IgG or non-reconstituted NSG mice treated with an anti-PD-1 antibody. Both tumors had similar immune cell infiltrations as determined by IHC staining. In one other case of metastatic melanoma (451Lu) that is aggressively growing in Hu-mice, treatment with anti-PD-1 had a negligible effect on tumor growth (Supplementary Fig. 8). No immune infiltrating cells were detected by IHC staining in 451Lu melanoma cells. In patients, tumor burden is a limiting factor to anti-PD-1 therapy responses^24^. Similar to patients’ responses, our results in Hu-mice also demonstrate heterogeneous responses to antibody therapy.

### Heterogeneous infiltration of T cells in tumors after anti-PD-1

**Fig. 3:**
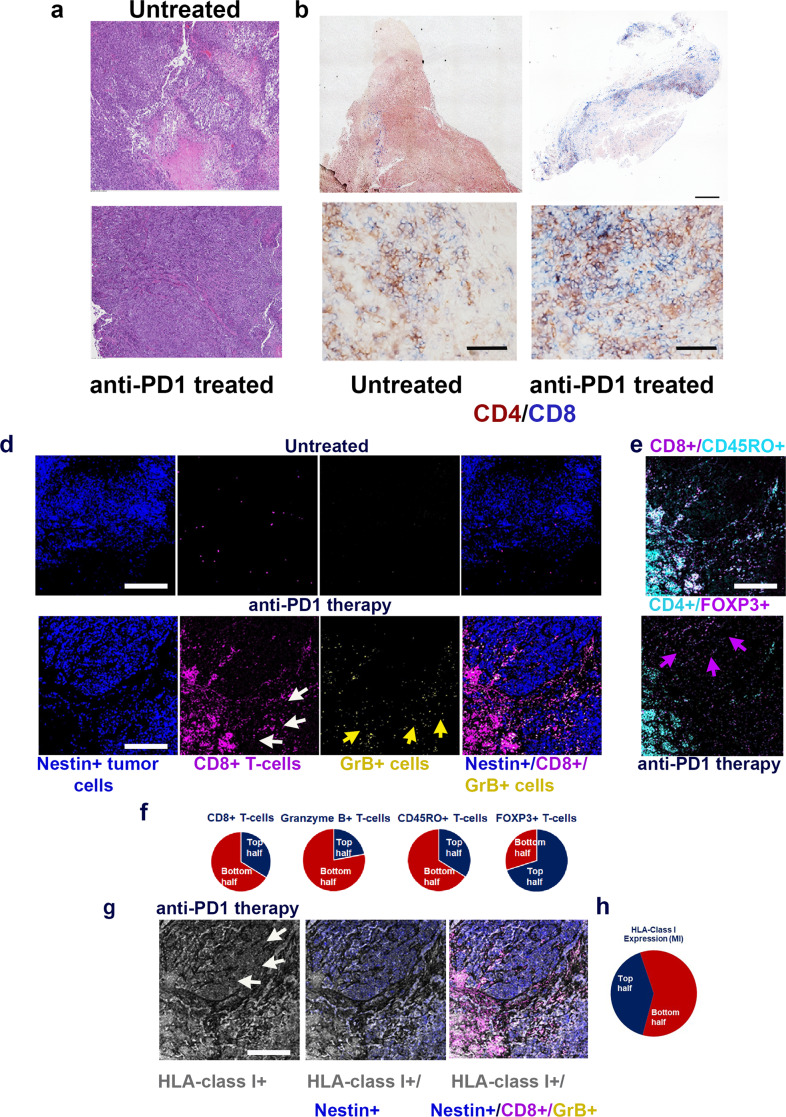
Immune and tumor heterogeneity as a possible cause of therapy resistance to anti-PD-1. **a**–**d** Heterogeneous distribution of leukocytes and immune cells in tumors after PD-1 treatment. a. Tumor (A375) bearing Hu-mice that received anti-PD-1 as in Fig. 2h, showed dense leukocyte infiltration of leukocytes (bottom panel; scale bar: 500 μm) when compared to mice that received control mouse IgG (top panel; scale bar: 100 μm) as determined by H&E staining. **b** Tumor (A375) bearing Hu-mice that received anti-PD-1 showed a heterogeneous distribution of CD4^+^ and CD8^+^ T cells (stitched image right panel; scale bar: 500 μm) when compared control Ig treated Hu-mice that had a sparse distribution of T cells (right panel). Please see Supplementary Fig. 9 for digital quantification of CD4^+^ and CD8^+^ T cells. **c** Tumor (A375) bearing Hu-mice that received anti-PD-1 showed either low to moderate (left panel; scale bar: 50 μm) or robust (right panel; scale bar: 50 μm) tumor-infiltration of CD4^+^ (brown) and CD8^+^ (blue) T cells within the same tumor. **d** MassCyTOF staining shows heterogeneous and higher distribution of CD8^+^ T cells (magenta) within the nestin^+^ tumor (A375) cells (dark blue) in anti-PD-1 treated tumor-bearing mice (lower panels) as compared to low infiltration of CD8^+^ T cells (upper panels) in untreated Hu-mice (see **f** for quantification). Distribution of GrB^+^ T cells (yellow arrows; 2nd to right bottom panel) was heterogeneous as they were higher on the bottom half (digital quantification:131 counts) of the tumor section when compared to the remainder of other nestin^+^ tumor cell areas (digital quantification: 37 counts). **e** CD8^+^ T cells are of memory phenotype as they stain for CD45RO (light blue; top panel) and areas not infiltrated by CD8^+^ T cells reveal the presence of CD4^+^/FOXP3^+^ cells (magenta arrows; bottom panel). **f** Quantification of MassCyTOF images by ImageJ software indicates a significant increase (*p* = 0.0049) in CD8^+^ T cells and Granzyme B^+^ T cells; CD45RO^+^ memory T cells compared to untreated mice and increase in FOXP3^+^ Treg cells in the indicated top or bottom half panels. All histology and MassCyTOF staining were consistent and confirmed in replicates of 2. **g**, **h** Downmodulation of HLA class I (white arrows) was observed in tumor areas that were associated with higher FOXP3^+^ cells (*p* = 0.0351; see **e** bottom panel; a representative image). Confirmation by quantitative analysis of mean intensity (MI) of HLA-class I expression (**h**). A one-sided paired *t*-test was used for analysis when *p*-values are provided. Representative scale bars in **d**, **e**, **g** represent 50 μm. All observations (**a**–**e**, **g**) were consistent, were observed in technical replicates (CyTOF), confirmed in histology staining, and in replicate experiments.

Next, to understand the phenomenon of mixed therapy responses to anti-PD-1 treatment, we performed IHC, multiplexed imaging using MassCyTOF, and RNA-seq on tumors obtained from anti-PD-1 treated Hu-mice and compared them with control Ig treated mice. Higher levels of immune infiltration were observed in tumor sections of anti-PD-1 treated Hu-mice when compared to untreated controls (see IHC staining; Fig. 3a). In addition, there was a heterogeneous distribution of CD4^+^ and CD8^+^ T cells within the tumors of mice that received anti-PD-1 therapy (Fig. 3b, c and Supplementary Fig. 9). Some regions of the tumors revealed poor infiltration of CD8^+^ T cells and this may have given rise to a therapy-resistant tumor that continued to show unrestricted growth. Multiplex imaging of tumor tissue sections by MassCyTOF with a panel of 25 rare earth metal-tagged antibodies revealed significantly higher levels of CD8^+^/Granzyme (Gr) B^+^ T cells (Fig. 3d–f) as quantified by using ImageJ software and represented as pie charts. CD8^+^/Granzyme (Gr) B^+^ T cells were of an effector memory phenotype (CD45RO^+^; Fig. 3e [top panel]) in mice that received anti-PD-1 treatment. There was a selective distribution of CD8^+^/Granzyme (Gr) B^+^ T cells (Fig. 3d, bottom 2 right panels). Further, there was an increased presence of FOXP3^+^ Treg cells in areas that lacked CD8^+^ T-cell infiltration (Fig. 3e [bottom panel]), and the same areas also had significant downmodulation of HLA class I expression as indicated by differences in mean intensity levels (*p* = 0.035099; Fig. 3g [left panel, white arrows] and Fig. 3h). These observations were similar in another sample that was used for CyTOF staining. Our observation is further supported by a finding that FOXP3^+^ upregulation results in repression of HLA class I molecules in epithelial cancer^10^. To dwell deeper into the mechanism of selective downmodulation of HLA class I expression, we performed RNA-seq analysis of tumors from Hu-mice treated with and without anti-PD-1 antibody.

### Tumor-infiltrating mast cells after anti-PD-1 treatment

**Fig. 4:**
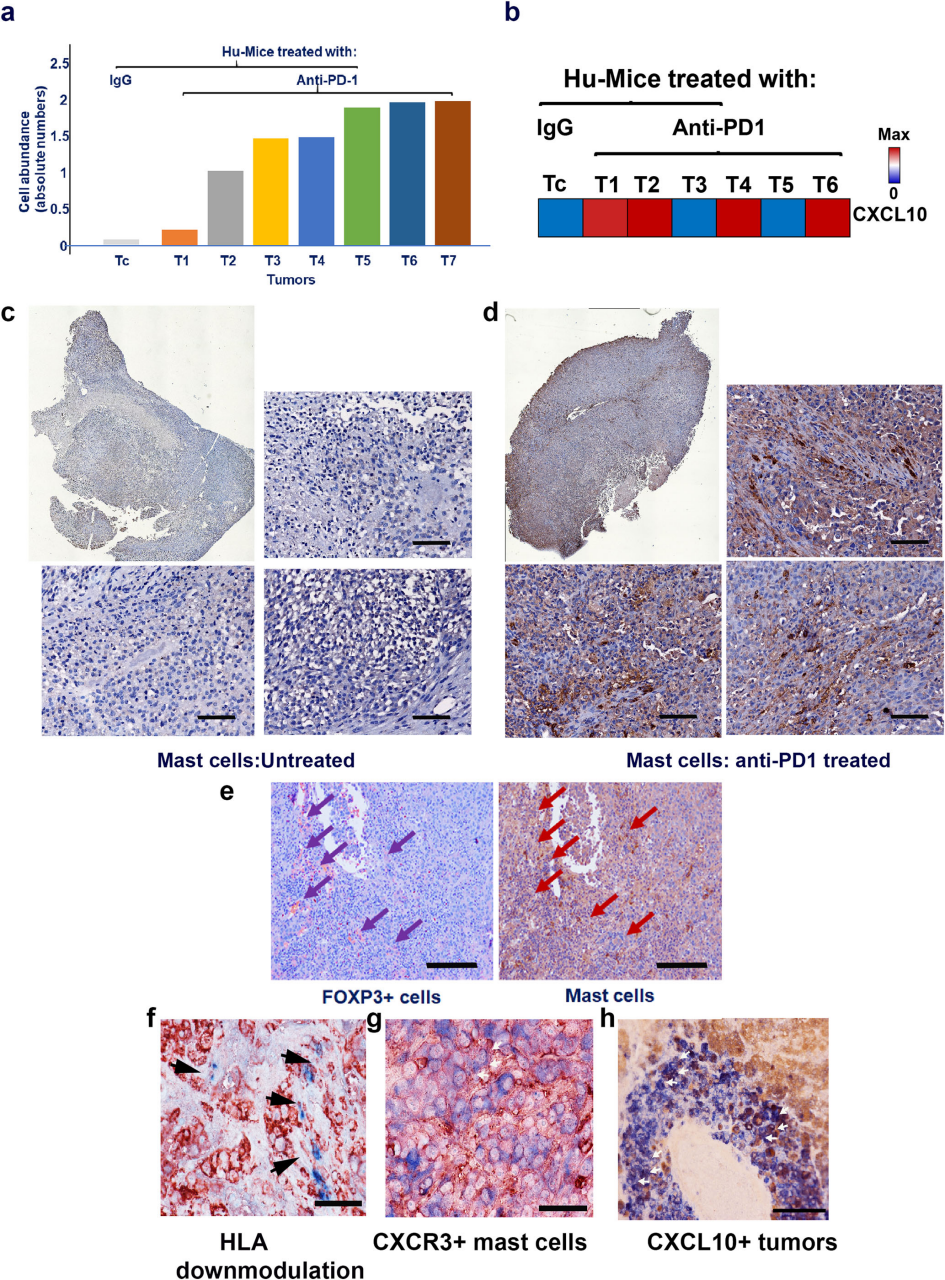
Increase in mast cells after anti-PD-1 therapy. **a** CIBERSORT analysis of the RNA-seq data set (GSE161353) showed a higher abundance of mast related genes in tumors (WM3629 and A375) obtained from Hu-mice after anti-PD-1 treatment and increased expression of CXCL10, a chemoattractant for mast cells (**b**). The presence of mast cells was further confirmed by mast cell tryptase IHC staining. **c**, **d** Stitched image (top left panels) and three different fields from an untreated tumor (WM3629) had negligible staining for mast cells (top right panel and bottom two panels; scale bar: 100 μm), whereas stitched image (top right middle panel) and three different fields from anti-PD-1 treated tumor had robust staining for mast cells, which was highly significant compared to control-treated mice (*p* = 0.005407; top right panel and bottom 2 panels; scale bar: 100 μm). **e** Co-localization of FOXP3^+^ Treg and mast cells after anti-PD-1 therapy. Co-localization of these cells as determined by IHC staining was significant (*p* = 0.000047; scale bar: 200 μm) suggesting crosstalk. **f** Downmodulation of HLA class I. HLA class I molecules as determined by staining with anti-HLA class I antibody (red) were downmodulated in tumor (WM3629) areas (black arrows) that were infiltrated by mast cells (blue). Scale bar represents 50 μm. **g** Mast cells co-express CXCR3. Mast cells were co-stained with anti-MCT (blue) and anti-human CXCR3 (red; white arrows) antibodies (see Supplementary Fig. 11 for digital quantification). Scale bar represents 50 μm. **h** Melanoma (WM3629) cells co-express CXCL10. Tumor cells were co-stained with anti-melanoma (HMB45 [blue]) and anti-human CXCL10 (red; white arrows) antibodies. Scale bar represents 100 μm. Histology staining (**c**–**h**) was confirmed in repeat experiments (2×). Source data are provided as a Source Data file (data are included as part of Supplementary Fig. 10).

To determine the immune phenotypes within the tumor cells, CIBERSORT analysis^25^ of the RNA-seq data was performed and it revealed a higher abundance of tumor resident resting mast cells (Fig. 4a and Supplementary Fig. 10a) and increased CXCL10 expression (Fig. 4b) in multiple tumor samples obtained after anti-PD-1 treatment indicative of the possible role of chemokine in the mobilization of these cells. Immune histology staining of mast cells confirmed significantly increased levels in Hu-mice tumors that received anti-PD-1 therapy when compared to control Ig treated mice (*p* = 0.005407; Fig. 4c, d). CIBERSORT analysis revealed other types of immune cell types including monocytes/macrophages, T- and B-cells in anti-PD-1 treated tumors, and the level of expression of these cells was heterogeneous between tumors (Supplementary Fig 10a). Gene expression related to neutrophils were relatively low in all tumors (Supplementary Fig 10a). Further examination of the tumor tissue sections after anti-PD-1 therapy revealed the co-localization of mast cells and FOXP3^+^ Treg cells, which was consistently observed and highly significant (*p* = 0.000047) when compared to control-treated tumors (Fig. 4e) and this may be associated with downmodulation of HLA class I on tumor cells (Fig. 3g, h, f). For the mast cells to be recruited by tumor cells, they need to express chemokine receptors^26,27^, one of them being CXCR3 (Fig. 4g), a majority of the tumor-infiltrating mast cells co-express CXCR3 (Supplementary Fig. 11), this was consistent in one another tumor sample. Melanomas are known to secrete CXCL10^28^, and we do confirm its presence in tumors (Fig. 4h). CXCL10 is known to bind to CXCR3, which is also present on mast cells^29,30^ and it is one of the probable reasons for increased infiltration of these cells into the tumor area.

### Infiltration of mast cells after immune-checkpoint therapy

**Fig. 5:**
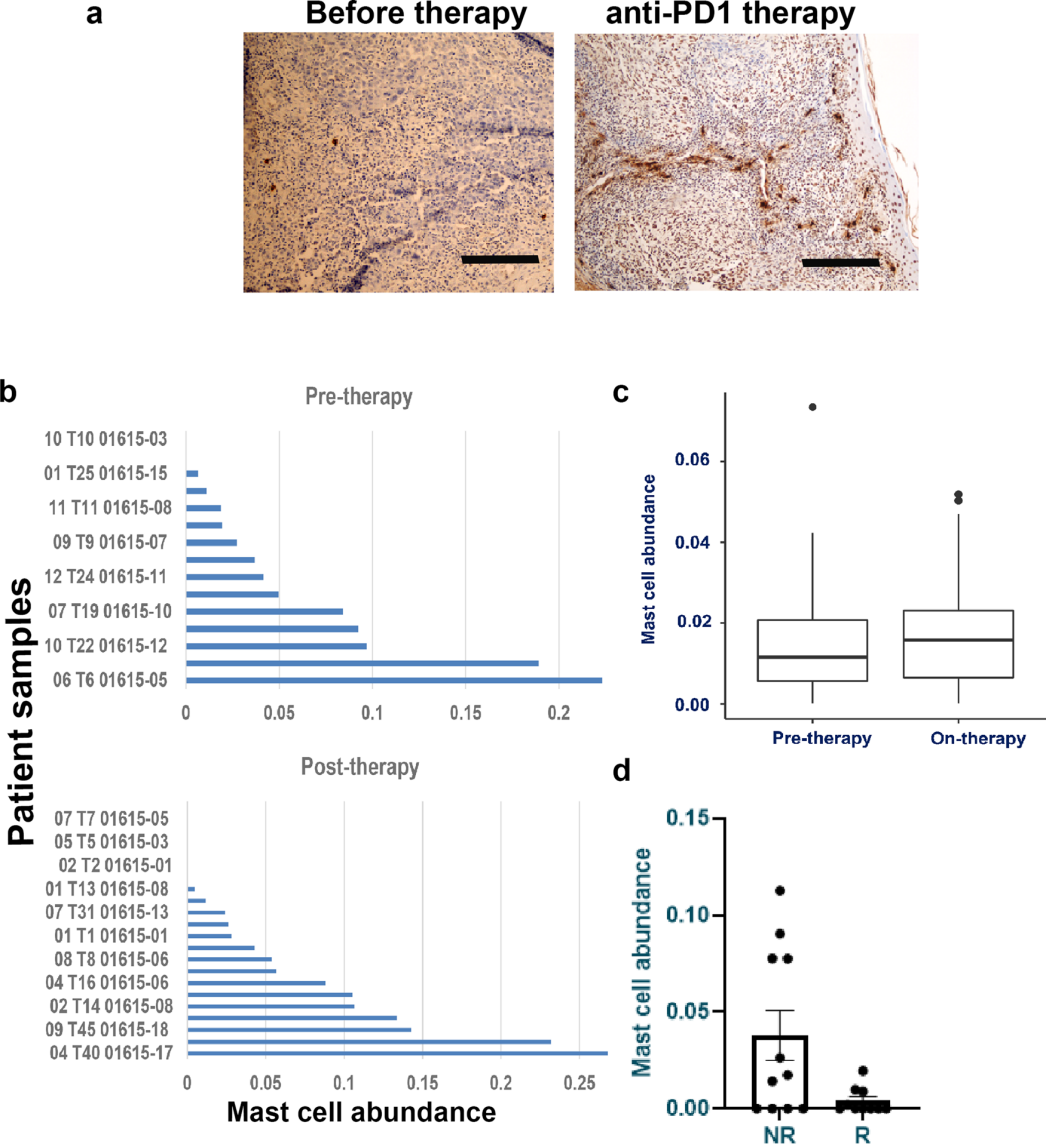
Anti-PD-1 therapy response is modulated by the presence of tumor-infiltrating mast cells. **a** Increase in mast cells in melanoma patients’ tumors after anti-PD-1 therapy. Immunostaining of human melanoma patients’ tumor showed an increased presence of mast cells after anti-PD-1 therapy (right panel) when compared to untreated individuals (left panel). Scale bars in both panels represent 200 μm. A representative staining is shown. **b**, **c** CIBERSORT analysis of three independent data sets (GSE123728 [16 pre and 23 on-therapy patients] and GSE91061 [43 pre-/on-therapy patients]) obtained from melanoma patients undergoing immune-checkpoint therapy showed a higher abundance of mast cell-related genes when compared to pre-therapy tumors and this was significant in **b** (*n* = 38; *p* = 0.0007) and **c** (*n* = 73; *p* = 0.049) due to higher number of matched pair biopsies (before and on-therapy samples). Box plots for pre-therapy tumors are represented as median (0.01153732; quartile 0.01153734), minimum (0; quartile 0.00561458), and maximum (0.07344924; quartile 0.02074330), and for on-therapy tumors are represented as median (0.01577157; quartile 0.01577157), minimum (0; quartile 0.00651041) and maximum (0.05185949; quartile 0.02311874). **d** Increased levels of mast cells in immune-checkpoint therapy non-responders. CIBERSORT analysis of RNASeq data set from MD Anderson trial (pre- and on-therapy patients (*n* = 23; Helmink et al.^32^)) revealed higher mast cell levels in anti-PD-1 therapy non-responder population. However, this increase was not significant due to smaller patient numbers. Data are presented as mean values ± SEM. A one-sided paired *t*-test was used for analysis when *p*-values are provided. Source data are provided as a Source Data file (data are included as part of Supplementary Fig. 10).

To understand the clinical relevance, the presence of mast cells was confirmed in tumor sections and in CIBERSORT analysis of three independent RNASeq data sets of melanoma patients receiving anti-PD-1 or immune-checkpoint therapies^12,31^ (Fig. 5a–d). The abundance of resting mast cells was higher and statistically significant in two clinical trial patients receiving immune-checkpoint inhibitors (*p* = 0.00001; GSE123728 and *p* = 0.049; GSE91061). In one another trial^32^, there was an increased abundance of mast cells in non-responder patients when compared to patients responding to anti-PD-1 therapy. However, due to the smaller number of patients enrolled in this study the difference was not statistically different. Besides tumor-infiltrating resting mast cells, other immune cell subtypes were observed in the tumor tissues. As before, the distribution of other immune cell subtypes in tumors was heterogeneous (Supplementary Fig. 10b–d).

### Depletion of mast cells improves anti-PD-1 treatment

**Fig. 6:**
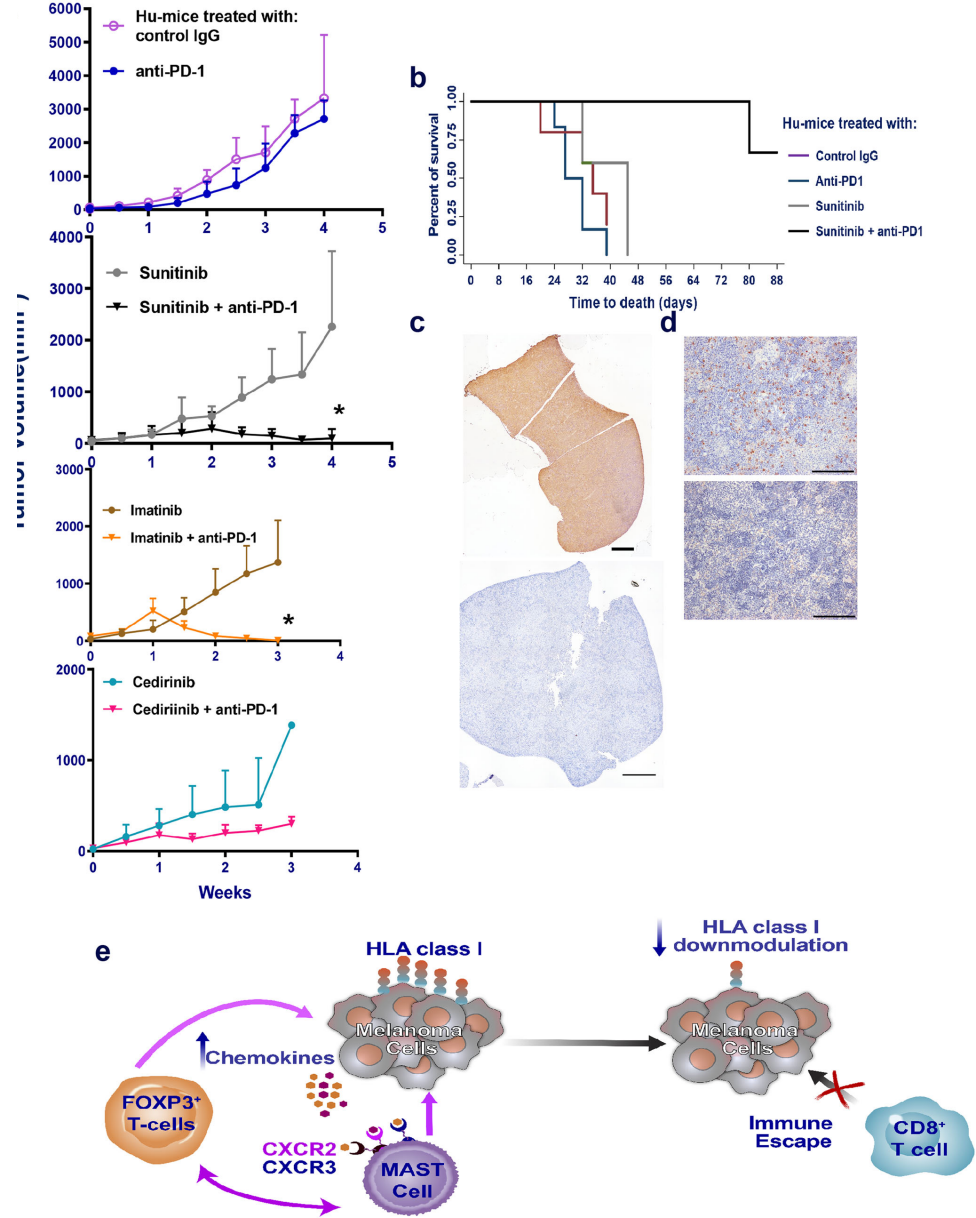
Complete regression of tumors after a combination of sunitinib and anti-PD therapy. **a** Using an independent batch of Hu-mice, established tumors (A375) (details as in Fig. 2g, h; *n* = 5/group) were treated with sunitinib (20 mg/kg) daily by oral gavage and after 72 h, anti-PD-1 therapy (10 mg/kg) was given weekly for a total of 6 injections. Complete tumor regression was observed in presence of combination therapy (black inverted triangle; *p* = 0.0001; 2nd panel), while sunitinib alone (gray circles), anti-PD-1 alone (blue line, closed circles) or control IgG (magenta; open circles) did not have any effect of tumor growth. We observed similar results when drug imatinib (50 mg/kg; daily by oral gavage) was used in combination with anti-PD-1 (brown line, closed circles; *p* = 0.0282; *n* = 7; 3rd panel from the bottom). Imatinib alone had no effect on tumor growth. Cediranib (6 mg/kg; daily by oral gavage) either alone or in combination with anti-PD-1 antibody was unable to shrink the tumors (*n* = 3 per group; bottom panel). Data are presented as mean values ± SD. A one-sided paired *t*-test was used for analysis when *p*-values are provided. **b** Survival curve from the above-treated mice indicates significant (*p* = 0.0013 as determined by the log-ranked test) survival advantage of tumor-bearing Hu-mice that received combination therapy of sunitinib and anti-PD-1 group when compared to sunitinib alone (gray), or anti-PD-1 alone (dark blue) or control IgG (magenta) groups**. c**, **d** Depletion of mast cells in spleen in sunitinib treated mice (bottom [1000] μm left and right panels [100] μm) when compared to control mice (top left [1000] μm and right panels [100] μm panel). Histology staining (**c**, **d**) was confirmed in repeat experiments (2×). **e** Schema of mast cell-induced resistance mechanism to anti-PD-1. Source data are provided as a Source Data file.

If mast cells are associated with therapy resistance of anti-PD-1 treatment, then their depletion should result in altered tumor growth. Mast cells are known to be c-kit receptor-positive and one can target these cells by pharmacological intervention using small molecule inhibitors. We used sunitinib, a multi-targeted receptor tyrosine kinase inhibitor including c-kit receptor that is known to deplete mast cells and Treg cells^33,34^ and followed it with anti-PD-1 therapy in treating established tumors in Hu-mice. Inclusion of sunitinib in combination with anti-PD-1 caused complete regression of tumors in 3/5 mice while treatment with sunitinib alone did not influence tumor growth significantly (Fig. 6a). Tumor-bearing Hu-mice receiving combination therapy of sunitinib and anti-PD-1 treatment survived longer than 48 days when compared to Hu-mice receiving sunitinib or anti-PD-1 or control IgG group (Fig. 6b). We have used imatinib, a similar drug to sunitinib but more specific for c-kit receptor, and found complete regression of tumors when Hu-mice received combination treatment of imatinib and anti-PD-1. Imatinib alone had no effect on tumor growth. Meanwhile, cediranib, a drug targeting VEGF and PDGF receptors failed to cause complete regression of tumors when included with anti-PD-1 therapy. This was not surprising as the melanoma cell lines used in the experiment have low to moderate expression of VEGF or PDGF receptors. Spleens from Hu-mice showed depletion of mast cells when compared to spleens from control Hu-mice that further confirms results from other groups on the action of sunitinib^33,34^ (Fig. 6c, d). Hu-mice that showed complete regression of tumors showed no signs of recurrence for 4 weeks after cessation of therapy and all the Hu-mice were able to reject re-challenged tumors indicative of memory T-cell responses. Our results suggest that mast cells are associated with therapy resistance to anti-PD-1.

## Discussion

Tumors play a dynamic role in evading therapy responses. This is done either directly or indirectly by enlisting the help of tumor stromal cells in therapy resistance^1,4^. Several mechanisms have been identified including alteration and/or activation of redundant signaling pathways, and downmodulation of the antigen-presenting machinery to evade anti-tumor-specific T cells, all contributing to the resurgence of resistant tumor cells^1,4^. In a recent study, TGFβ has been identified as a major player in driving a resistant phenotype and contributing to MHC class I downmodulation in melanoma^35^. We and others have shown that tumor-infiltrating fibroblasts, macrophages, and B-cells have an important role in therapy resistance^2,36,37^. Tumor-infiltrating mast cells have been implicated in therapy resistance and tumor progression in breast and prostate cancer^38,39^.

In the present study, our results suggest that the presence of tumor-infiltrating mast cells and FOXP3 cells is associated with downmodulation of HLA-class I on tumor cells, lack of CD8^+^ T cells in these areas, and in effective tumor killing and eventual immune escape after anti-PD-1 therapy (Schema Fig. 6e). There is an increase in chemokine production causing increased infiltration of mast cells into the tumor after anti-PD-1 therapy. Co-localization of mast cells and FOXP3^+^ Treg cells was observed in selective areas of the tumor sections presumably causing localized pockets of resistance. The co-presence of FOXP3^+^ Treg cells and mast cells is associated with lower expression of HLA-class I in tumors (Fig. 3e, f, h). Lower expression of HLA-class I on melanoma cells is associated with poor infiltration of CD45RO^+^, CD8^+^, Granzyme B^+^ T cells and hence probable cause of tumor escape, and therapy resistance. The combination of sunitinib or imatinib, both inhibitors of receptor tyrosine kinases including c-kit, and anti-PD-1 resulted in complete regression of tumors suggesting that depletion of mast cells is beneficial to immune-checkpoint therapy responses. Association of mast cells with resistance to anti-PD-1 therapy warrants more investigation that will provide new direction and a rationale for developing new combined immune therapy approaches with small-molecule signaling inhibitors for the treatment of melanoma patients.

## Methods

### Reagents

A list of antibody reagents used in this study is provided in Supplementary Table 1. For a panel of metal conjugated antibodies used in multiplexed imaging of tissue CyTOF, please see Supplementary Table 2 and refer to Wang et al.^40^.

### Tissues and cell lines

Human melanoma tissues were obtained in accordance with informed consent procedures approved by the Internal Review Boards (IRB) of the Hospital of the University of Pennsylvania and The Wistar Institute, Philadelphia. Fetal liver and thymus tissues were obtained in accordance with informed consent that was approved by IRB of Advanced Bioscience Resources, Alameda, CA, and The Wistar Institute. Human melanoma cell lines (A375, 451LU, WM3629) have been described in detail earlier^41^ and in brief, they were isolated from patients’ tumor tissue and established as cell lines that are cultured in DMEM or RPMI 1640 medium supplemented with l-glutamine and 5% FBS. All cell lines were tested for mycoplasma and short tandem repeat profile (DNA identity) before being used for any experiments.

### In vivo mouse studies

All animal experiments were performed according to protocols approved by the Wistar Institute’s Institutional Animal Care and User Committee (IACUC). NOD/LtS*scid*IL2Rγ^null^ (NSG) mice were inbred at The Wistar Institute under license from the Jackson Laboratory (00557). All studies were performed in accordance with the Guide for Care and Use of Laboratory Animals published by the U.S. National Institute of Health. Wistar Animal Facility is fully accredited by the Association for Assessment and Accreditation of Laboratory Animal Care (AAALAC) and houses rodents for experimental research. Mice were housed in temperature-controlled holding rooms (21–22 °C), 30–34% humidity, and on a 12 h on (6 AM) and off light cycle (6 PM). They are housed in well ventilated disposable cages with irradiated corn bedding and individually ducted Innovive racks. Both experimental and control animals were kept in the same holding room, handled aseptically in germ-free environment in Biosafety Cabinet (Class II), socially caged as 5 mice/unit cage, and fed with irradiated food pellets and HCL water. Wistar Animal Facility has a quality control program in place wherein 5% of mice in each holding room were periodically tested serologically for common murine viruses (Sendai, pneumonia virus of mice, mouse hepatitis virus, theoloviruses, reo virus, lymphocyte choriomeningitis virus, ectromelia virus, mouse pneumonitis virus, polyoma virus, mastadeno virus [1 and 2], mouse rotavirus, mouse parvovirus, and murine Nora virus), mycoplasma pulmonis and helicobacter. Sentinels from each rack in the holding room are sent to Charles River (Malvern, PA) for diagnostic testing. All mice were monitored three times a week by veterinarians. For humanization, fetal liver and thymus were obtained from the same donor (18–22 weeks of gestation). Female NSG mice (6–8 weeks) received thymus graft (1–2 mm^3^) in sub-renal capsule 24 h post myeloablation using Busulfan (30 mg/kg, i.p.; Sigma-Aldrich [B2635], St. Louis, MO). This is immediately followed by injection of autologous liver-derived CD34^+^ hematopoietic stem cells (10^5^ cells/mouse, i.v.; Fig. 1a) that was magnetically sorted by microbeads conjugated with anti-human CD34^42^ (Miltenyi Biotec., [130-046-703], Auburn, CA). Six to 8 weeks (>50 days) later, the presence of human immune cells was monitored by multi-color flow cytometry using 18 color BD LSR II Analyzer^2^ (BD Biosciences). To accelerate human immune cell reconstitution mice received AAV8 encoding human IL-3, IL-7, and GM-CSF^43^ (lysates from hepatocyte cell line infected with this AAV8 showed 23–40 pg/ml of cytokines) 6–7 days after CD34^+^ cell injections (Fig. 1c and Supplementary Fig. 1a; 10^8^; iv). All mice from Fig. 1d received also received DNA plasmid delivery (electroporation of anterior tibialis muscle; 50–100 μg DNA) of constructs encoding FLT3, SCF, THPO (Supplementary Fig. 1b). DNA plasmid delivery was after 6–7 days after AAV8 human cytokine injections. Serum obtained from mice that received DNA plasmids showed 20–60 pg/ml of the above cytokines for 3–4 weeks and gradually tapering off after 4 weeks. Six to 8 weeks (>50 days) later, the presence of human immune cells was monitored by multi-color flow cytometry using 18 color BD LSR II Analyzer^2^ (BD Biosciences). For this, 100 μl blood from submandibular bleeding was collected in BD Microtainer blood collection tubes that are coated with lithium and heparin. Red blood cells were lysed using ACK lysis buffer (Life Technologies Corporation, Carlsbad, Ca) followed by one wash using FACS medium (Phosphate buffered saline, 2% FBS, and 0.1% Na azide) then resuspended in 100 μl medium and incubated with a panel of fluorochrome-conjugated cocktail antibodies (Supplementary Table 1) for 30 mins. Excess antibodies were removed by washing once and then, cells were resuspended in 300 μl FACS medium for flow analysis to determine the percentage of human immune cell types. CountBright absolute counting beads (Life Technologies) were used for obtaining absolute lymphocyte counts using the manufacturer’s protocol. To distinguish between live and dead cells, cells were stained with DAPI (MP Biomedicals, Solon, OH). Cells were stained with anti-mouse CD45 and anti-human CD45 antibodies to distinguish between mouse and human cells and all the human subpopulation was gated on human CD45^+^ cells (see Supplementary Fig. 12 for gating strategy). Mice were considered humanized if human CD45 reached ~25% or more (650 to 800 cells/μl) in the peripheral blood of animals. All reconstituted mice were assigned into experimental groups according to the levels of human immune cells (CD45^+^/CD8^+^ cells; 90 to 120 cells/μl). Mice were subcutaneously injected with HLA-A allele melanoma cells (10^5^) over the right flank. All tumors were treated once they became palpable (~100 mm^3^) with anti-PD-1 (10 mg/kg; 1× weekly; 5–6 injections; Keytruda, Merck, Rahway, NJ) antibody and respective IgG antibody was used as control at similar dosage and frequency. Select groups of mice received sunitinib (20 mg/kg daily), imatinib (50 mg/kg, daily), or cediranib (6 mg/kg, daily); all drugs delivered as oral gavage and obtained from Cancer Therapy Evaluation Program ([CTEP], NCI, Bethesda, MD) or used in combination with an anti-PD-1 antibody for 5–6 weeks. Hu-mice that showed complete regression of tumors were given a drug holiday of 4 weeks and then re-challenged with half the number of same tumor cells. Hu-mice with tumors in excess of 500 mm^3^ were monitored daily and mice showing tumor necrosis or tumor size exceeding 2000 mm^3^ were euthanized according to Wistar IACUC guidelines. Tumors were measured twice a week using digital calipers.

### HLA typing

Fetal liver or melanoma cell genomic DNA was isolated using a GeneJET genomic DNA purification kit (ThermoFisher, [K0722], Waltham, MA). Standard PCR was performed using the following HLA allele-specific primers purchased from Integrated DNA Technologies (Coralville, IA): HLA-A1 forward 5′-ACA GAC TGA CCG AGC GAA and reverse 5′-CTC CAG GTA GAC TCT CCG; HLA-A2 forward 5′-GAC GGG GAG ACA CGG AAA and reverse 5′-CAA GAG CGC AGG TCC TCT; HLA-A3 forward 5′-CGG AAT GTG AAG GCC CAG and reverse 5′-CAC TCC ACG CAC GTG CCA; HLA-A9 forward 5′-CAC TCC ATG AGG TAT TTC TC and reverse 5′-CAA GAG CGC AGG TCC TCT; b2 micro-globulin forward 5′-CGA TAT TCC TCA GGT ACT and reverse 5′-CAA CTT TCA GCA GCT TAC; b-actin forward 5′-TGC TAT CCC TGT ACG CCT CT and reverse 5′-CCA TCT CTT GCT CGA AGT CC. PCR cycling conditions were as follows: an initial denaturation step at 95 °C for 5 min and 30 cycles of denaturation (95 °C, 30 s), annealing (56 °C, 30 s), and extension (72 °C, 30 s) followed by a final extension at 72 °C for 10 min^44^. The HLA A locus sequence was determined using the SeCore kits (One Lambda) and Applied Biosystems 3130xl Genetic Analyzer (ThermoFisher). HLA sequence analysis software (uType Dx) was used for analysis and allele assignment.

### Immunostaining

IHC staining was performed as previously described^2^. Briefly, tissue sections were subjected to antigen retrieval by incubation with Target Retrieval Solution (Citrate [S1699] or Tris-EDTA buffers [S2367]; Agilent-DAKO, Santa Clara, Ca) kit at 95 °C for 20 min and subsequently incubated with primary antibodies with optimum dilutions (FOXP3 [1:10]; CD4, HLA class I, HMB45 and mast cell tryptase [all at 1:100 dilution]; CD68 [1:400 dilution] and CD8 [1:500 dilution]; TCR gamma/delta [1:50 dilution]). For detection of primary antibodies, slides were incubated with anti-mouse, anti-rat, or anti-rabbit antibodies at 1:1000 dilution and visualized by DAB (SK-4100) or AEC (SK-4200; both Vector Laboratories, Burlingame, CA) chromogens. Binding of mouse anti-human TCR γ/δ was visualized by using anti-mouse Qdot 625 at 1:500 dilution.

### Multiplexed tissue MassCyTOF staining

CyTOF staining was performed as previously described^40^. Briefly, carrier-free antibodies were commercially obtained (Supplementary Table 2) and tagged with lanthanide metals using the Maxpar X8 metal conjugation kit from Fluidigm^R^ (201300, Ontario, Canada). Antigen retrieval was performed on deparaffinized tissue sections at 95 °C for 30 min in Tris/EDTA buffer, slides were cooled, blocked with 3% BSA-PBS solution, and incubated with a cocktail of antibodies (100 μl) overnight at 4 °C. Next, day slides were washed 3× with PBS and labeled with 1:400 dilution of Intercalator-Ir (Fluidigm 201192B) in PBS for 30 min at RT. Slides were washed with water (3×) and air-dried for 30 min before image mass cytometry acquisition using Fluidigm Hyperion Imaging System.

### RNA-seq and CIBERSORT

RNA was isolated from spleen and tumor tissues and tissue blocks obtained from pre- and post-therapy (anti-PD-1) mice using Zymo Direct-zol RNA MiniPrep kit (Zymo Research, [R2073] Irvine, CA). Total RNA was subjected to ribosomal RNA depletion followed by library preparation using ScriptSeq Complete Gold Kit (BG1224; Illumina, Madison, WI), which uses the patented terminal-tagging process to generate directional libraries. Quality control of RNA and final libraries was done using the Tapestation 4200 and Bioanalyzer 2100 system (Agilent, Santa Clara, CA). Final libraries were quantified using the KAPA Library Quant qPCR kit (Roche, [KK4835] Pleasanton, CA) and subjected to 75 bp paired-end high output sequencing run on Illumina’s NextSeq500. RNA-seq data analysis was performed using RSEM v1.2.12 software and downstream expression analysis was done using DESeq2^45^. Normalized RNA-seq data was used to enumerate tumor-infiltrating leukocytes using CIBERSORT, an analytical tool available online^25^.

### Spleen T-cell stimulation

Spleen cells freshly harvested from the spleen of Hu-mice (5 × 10^4^ per well of a 96-well round-bottomed microtiter plate; Corning, NY) were stimulated for 72 h with various melanoma peptides (Supplementary Fig. S6 legend). All preparations were in the T-cell medium [RPMI 1640] with GlutaMAX medium (Life Technologies-Invitrogen, Carlsbad, CA) supplemented with 5% FBS. After the incubation, live cells were used for RNA extraction (see above) and used for cDNA synthesis using the Maxima First Strand cDNA synthesis kit (ThermoFisher).

### Real-time PCR

In brief, qPCR was performed using an Applied Biosystems’ 7500 Fast Real-Time PCR System with Power SYBR Green PCR Master Mix (Life Technologies). Custom made qPCR primers (Supplementary Table 3), were purchased from Integrated DNA Technologies (Coralville, IA). Thermal cycler conditions were 95 °C for 15 min and 40 cycles of 15 s at 95 °C followed by 1 min at 60 °C. All experiments were performed in triplicate, and the mean value was used for the determination of mRNA levels. The relative quantification (RQ) and expression of each mRNA were calculated using the comparative CT method according to the manufacturer (Applied Biosystems 7500 Software v2.0). All samples were normalized to an endogenous control, GAPDH.

### T-cell receptor (TCR) sequencing

TCR libraries for next-generation sequencing of the immune repertoire were generated using QIAseq Immune Repertoire RNA Library Prep. kit (IMHS-001Z Human TCR Panel, QIAGEN, Maryland, USA) which is designed to enrich TCR α, β, γ, and δ subunits. Briefly, RNA as isolated above from spleen and tumor tissues was used for TCR-specific cDNA synthesis, enrichment of complete TCR variable region by gene-specific primers targeting constant regions, and molecular indexing (UMIs) for accurate and sensitive TCR clonotype and repertoire diversity assessment. Final libraries were sequenced on Illumina’s NextSeq500 using the 300 cycles mid output sequencing kit. The clonotype calls were generated using the IMSEQ software^46^ through QIAGEN web-server (https://qiagen.com). Counts of UMIs supported by at least three reads were used as a measurement of relative TCR receptor sequences. Relative frequencies of unique V-J clonotypes were used to calculate diversity using Shannon–Wiener index and plotted as heatmaps.

### Grouping of lymphocyte interactions by paratope hotspots (GLIPH)

The GLIPH algorithm reveals TCR-antigen specificities shared between T-cell clonotypes^47^. In detail, it clusters TCR CDR3 sequences according to their local and global similarity, which are likely to react with the same peptide/MHC complex. A local similarity is defined by sequences containing the same specific motif of four amino acids, which is overrepresented in the analyzed data set compared to a large general naïve reference database; a global similarity refers to sequences having a Hamming mutation distance of one. The size of the circle reflects the relative number of detected TCR transcripts, i.e., the abundance of the respective T-cell clonotype. Moreover, to test which epitope/MHC a dominant TCR cluster is likely to be reactive with, we spiked in known TCR sequences recognizing HLA-A2-restricted melanocyte differentiation antigens (MDAs), Cancer Testis Antigens (CTA) or virus-derived epitopes (notably, these are depicted as rectangles with a fixed size). These respective TCR sequences were derived from the vdjdb database (https://vdjdb.cdr3.net/) or from a recently released 10× Genomics data set (https://support.10xgenomics.com/single-cell-vdj/datasets; “A New Way of Exploring Immunity”). For the execution of GLIPH and visualization of the results, the software R v3.4.1 was used.

### Statistics

Kaplan–Meier survival analysis and Student’s *t*-test were used. As software tools, R statistical package, GraphPad Prism, and Microsoft Excel were used.

### Reporting summary

Further information on research design is available in the Nature Research Reporting Summary linked to this article.

## Supplementary information

Supplementary Information

Reporting Summary

## Data Availability

RNAseq data sets (GSE161353) are available in the public domain. Other data generated and analyzed during the current study are available from the corresponding authors upon request. Source data are provided with this paper.

## Source data

Source Data
