# Supplementary material for: Tumor-infiltrating mast cells are associated with resistance to anti-PD-1 therapy
Source: Nat Commun. 2021 Jan 12;12:346. doi: 10.1038/s41467-020-20600-7 (PMC7804257; doi:10.1038/s41467-020-20600-7)
Supplement: Supplementary file 1 — Supplementary Information [file 41467_2020_20600_MOESM1_ESM.pdf]

Tumor-infiltrating mast cells are associated with resistance to anti PD 1 therapy.

Rajasekharan Somasundaram, Thomas Connelly, Robin Choi, Hyeree Choi, Anastasia Samarkina, Ling Li, Elizabeth Gregorio, Yeqing Chen, Rohit Thakur, Mohamed Abdel-Moshen, Marilda Beqiri, Meaghan Kiernan, Michela Perego, Fang Wang, Min Xiao, Patricia Brafford, Xue Yang, Xiaowei Xu, Anthony Secreto, Gwenn Danet-Desnoyers, Daniel Traum, Klaus H Kaestner, Alex Huang, Denitsa Hristova, Joshua Wang, Mizuho Fukunaga-Kalabis, Clemens Krepler, Fang Ping-Chen, Xiang Y Zhou, Alexis Gutierrez, Vito W Rebecca, Prashanthi Vonteddu, Farokh Dotiwala, Shashi Bala, Sonali Majumdar, Harsh Dweep, Jayamanna Wickramasinghe, Andrew V Kossenkov, Jorge Reyes, Kenisha Santiago, Tran Nguyen, Johannes Griss, Frederick Keeney, James Hayden, Brian J Gavin, David Weiner, Luis J Montaner, Qin Liu, Lukas Peiffer, Jürgen Becker, Elizabeth M Burton, Michael A Davies, Michael T Tetzlaff, Kar Muthumani, Jennifer A Wargo, Dmitry Gabrilovich and Meenhard Herlyn.

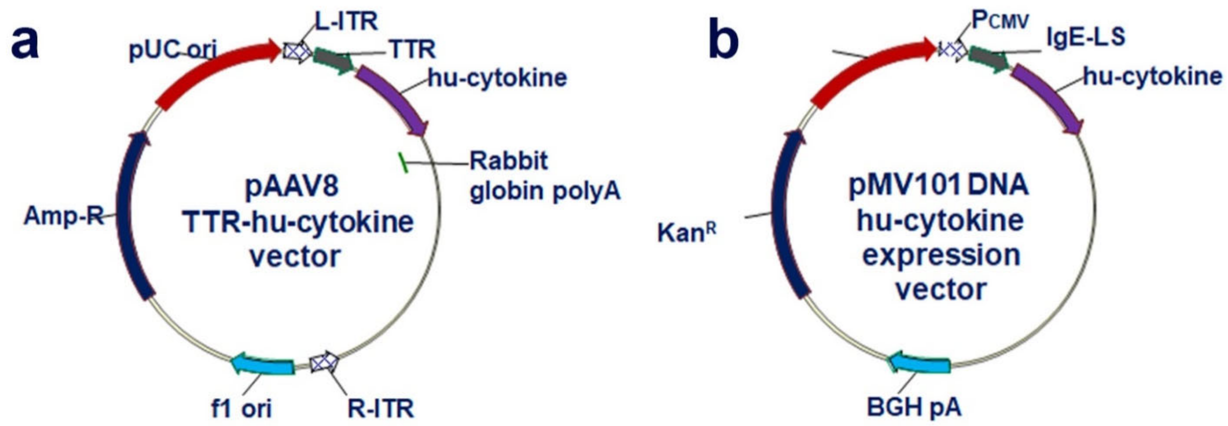

Supplementary Figure 1. Vector maps. Schema of AVV8 (**a**) and pMV101 (**b**) DNA encoding hu-cytokine transgenes. pMV101 is a modified pVAX1 vector[48].

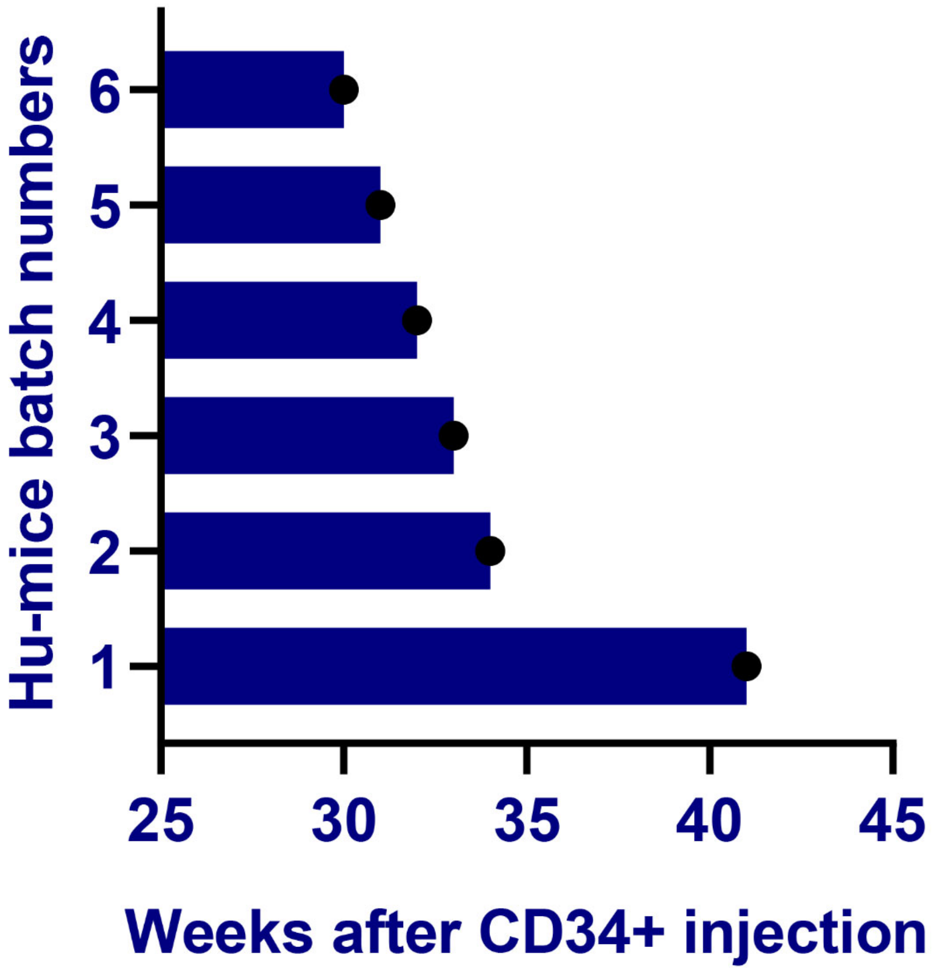

Supplementary Figure 2. Stability of Hu-mice. Representative examples of Hu-mice batches that received AAV8 and DNA plasmid encoded human cytokines as described in Figure 1 with longevity of 30 weeks or more after human $CD34^+$ cell injections.

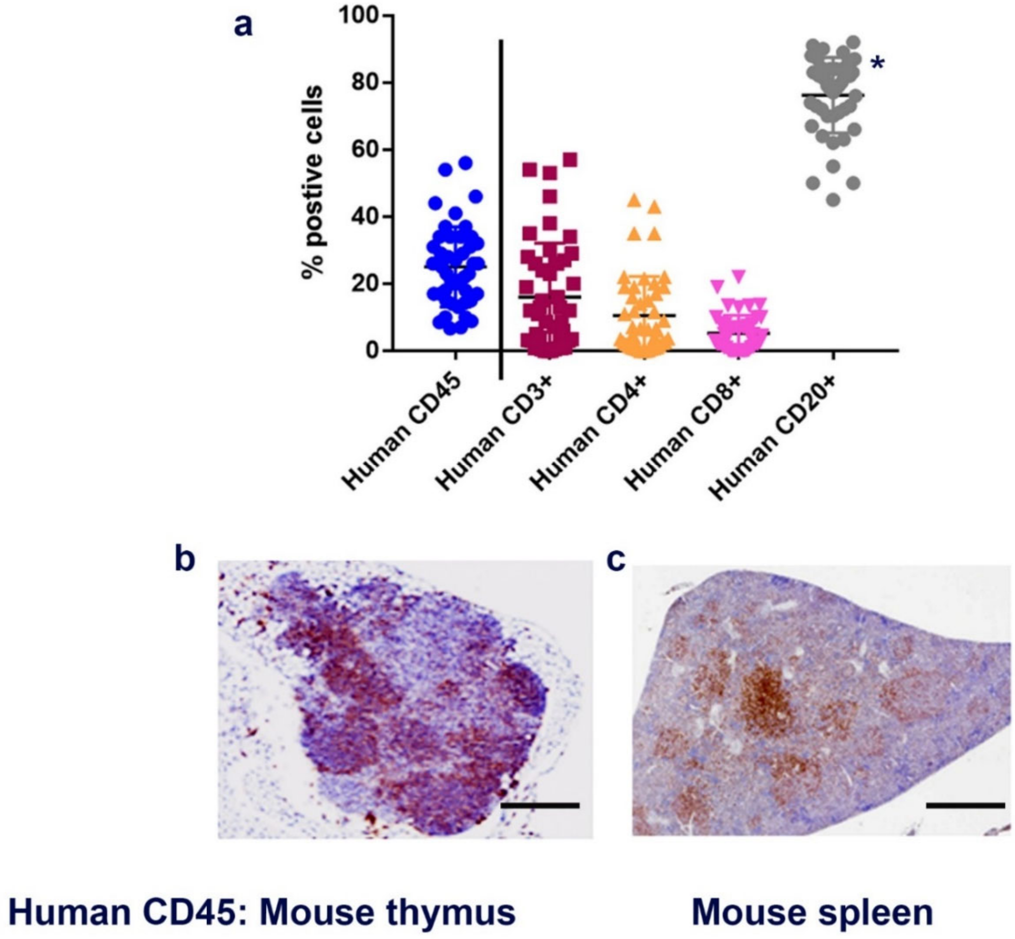

Supplementary Figure 3. Higher repopulation of human B-cells than T cells. Reconstituted humanized mice (n=45) as described in Figure 1 showed increased levels of B-cells than T cells (*p=0.000023) during early phase (8-10 weeks) of human lymphocyte reconstitution (**a**)**. b and c.** Human CD45$^+$ cells in reconstituted mouse thymus and spleen. Human CD45$^+$ cells (**brown staining**) are seen in lymphoid organs of mouse thymus (**b**) and spleen (**c**) as determined by IHC staining using anti-human CD45 antibody. All mice were euthanized by CO2 inhalation/cervical dislocation and organs harvested 24 weeks after CD34$^+$ cell injections. Scale bars in both **3b** and **3c** represents 250 µm. Histology staining (b, c) was confirmed in repeat experiments (2x). One sided paired t-test was used for analysis when p values are provided. Source data are provided as a Source Data file.

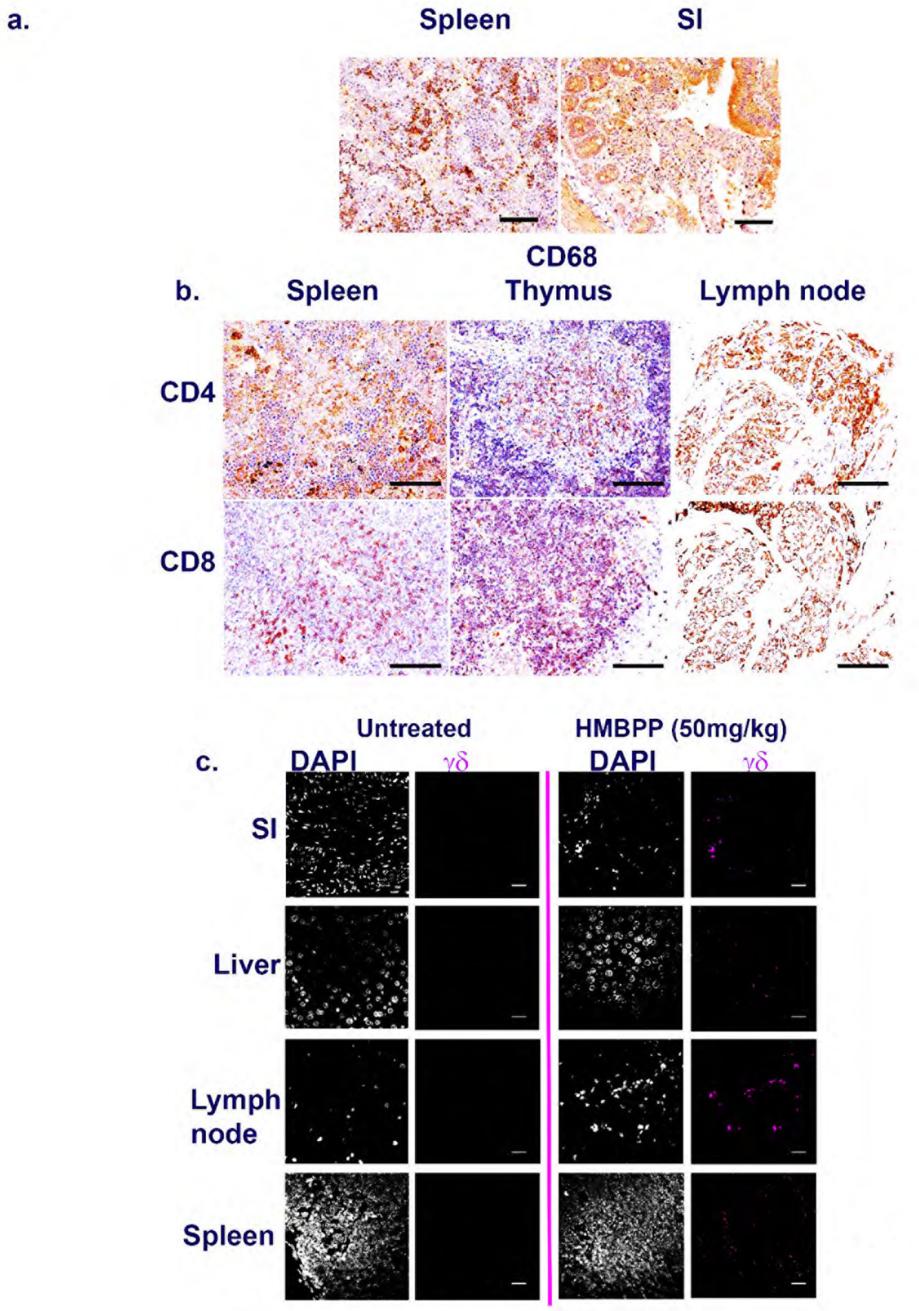

Supplementary Fig. 4. Human immune subpopulation in spleen, thymus, lymph node, small intestines (SI) and lungs of humanized mice. a. Human macrophages in the spleen and SI. Representative section from mouse spleen and SI shows the presence of $CD68^+$

monocyte/macrophage lineage cells **(left and right panels; scale bars: 200 μm)**. **b. Human $CD4^+$ and $CD8^+$ T-cell subpopulation in lymphoid organs.** Mouse spleen **(left panels; a)**, thymus **(middle panels)** and mesenteric lymph nodes **(right panels)** show presence of $CD4^+$ (**top panels**) and $CD8^+$ (**bottom panels**) T cells as determined by IHC staining using anti-human CD4 or CD8 antibodies. **Scale bars in all the panels represent 200 μm. c**. **Human Tγ/δ cells.** Reconstituted Hu-mice show presence of T γ/δ cells in the SI, liver, lymph node and spleen **(right most panels; scale bars: 25 μm)** of mice that were treated with a bacterial metabolite HMBPP at 50 mg/kg (i.p.). Presence of T γ/δ cells were determined in an IHC staining by using mouse anti-human TCR γ/δ primary antibody followed by detection using secondary anti-mouse Qdot 625 antibody. Histology staining (a-c) was confirmed in repeat experiments (2x).

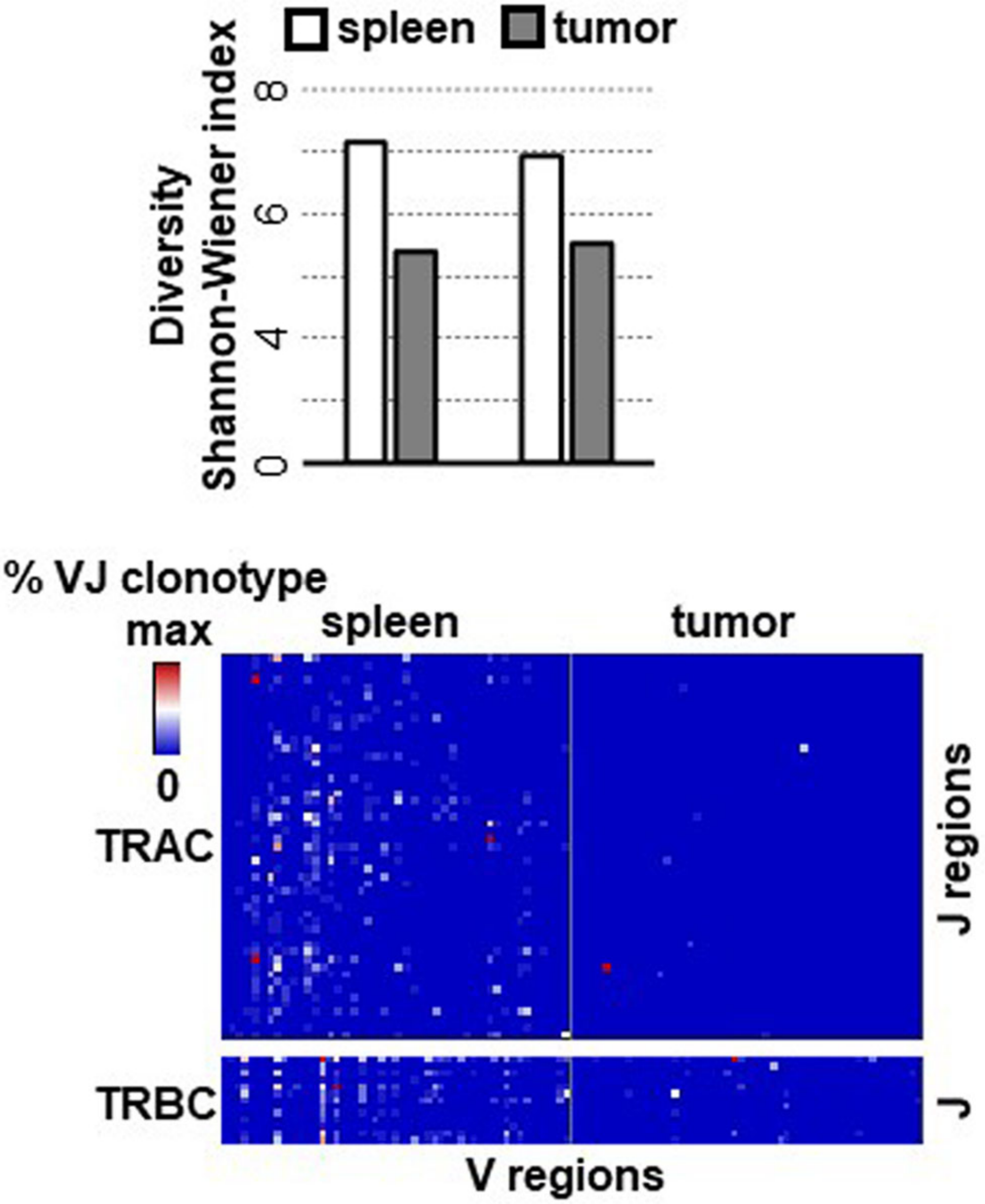

Supplementary Figure 5. Human Tα/β expression. TCR sequence analysis of spleen and tumor cells obtained from Hu-mice melanoma model showed more diverse expression of T α/β chains in the spleen when compared to more restricted usage in tumors (**top panel**). TCR α/β chain expression showed high prevalence of several unique VJ clonotypes in tumors (**bottom panel**).

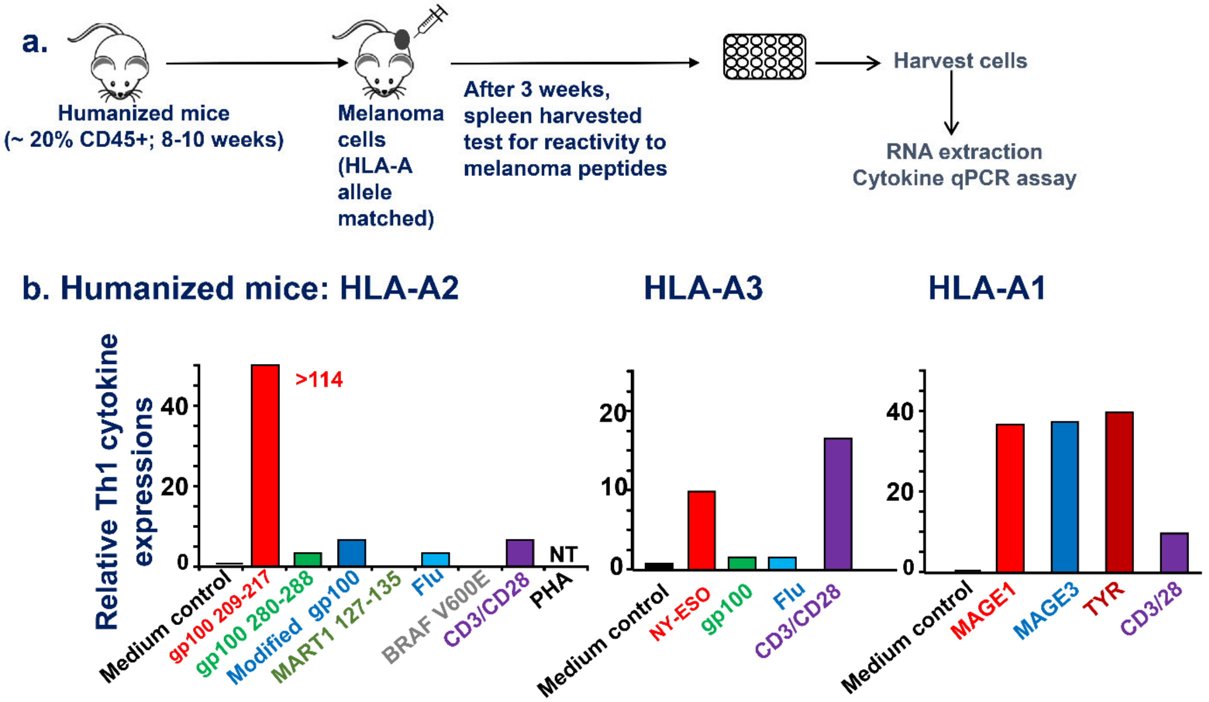

Supplementary Figure 6. Spleen T cells obtained from tumor challenged Hu-mice from 3 different batches of $CD34^{+}$ donors (HLA-A1, -A-2 and -A3) react to melanoma-specific T-cell peptide antigens after *in vitro* stimulation. a. Schema of tumor cell challenge and cytokine PCR. b. Spleen T cells show reactivity to melanoma T-cell peptide antigens as measured by real time PCR. Spleen T cells were stimulated *in vitro* for 72 h with various melanoma peptides (HLA A1 peptides, MAGE1: EADPTGHSY; MAGE3:EVDPIGKLY; TYR:KSDICTDEY; Flu: CTELKLSDY; HLA-A2 peptides, gp100$_{209-217}$ ITDQUPSV; gp100$_{280-288}$ YLEPGPUTA; mod gp100: IMDQVPFSV; MART:AAGIGILTV; HLA-A3 peptides, NY-ESO: ELARRSLAQ and SLLMWITQC [pooled], gp100: LIYRRRLMK, Flu: ILRGSVAHK; all at a concentration of 25μM) showed robust reactivity indicating sensitization to these antigens *in vivo* on tumor challenge. Assay was performed by real time PCR as described before[2] using standard Th1 cytokine primers (See Supplementary Table 3).

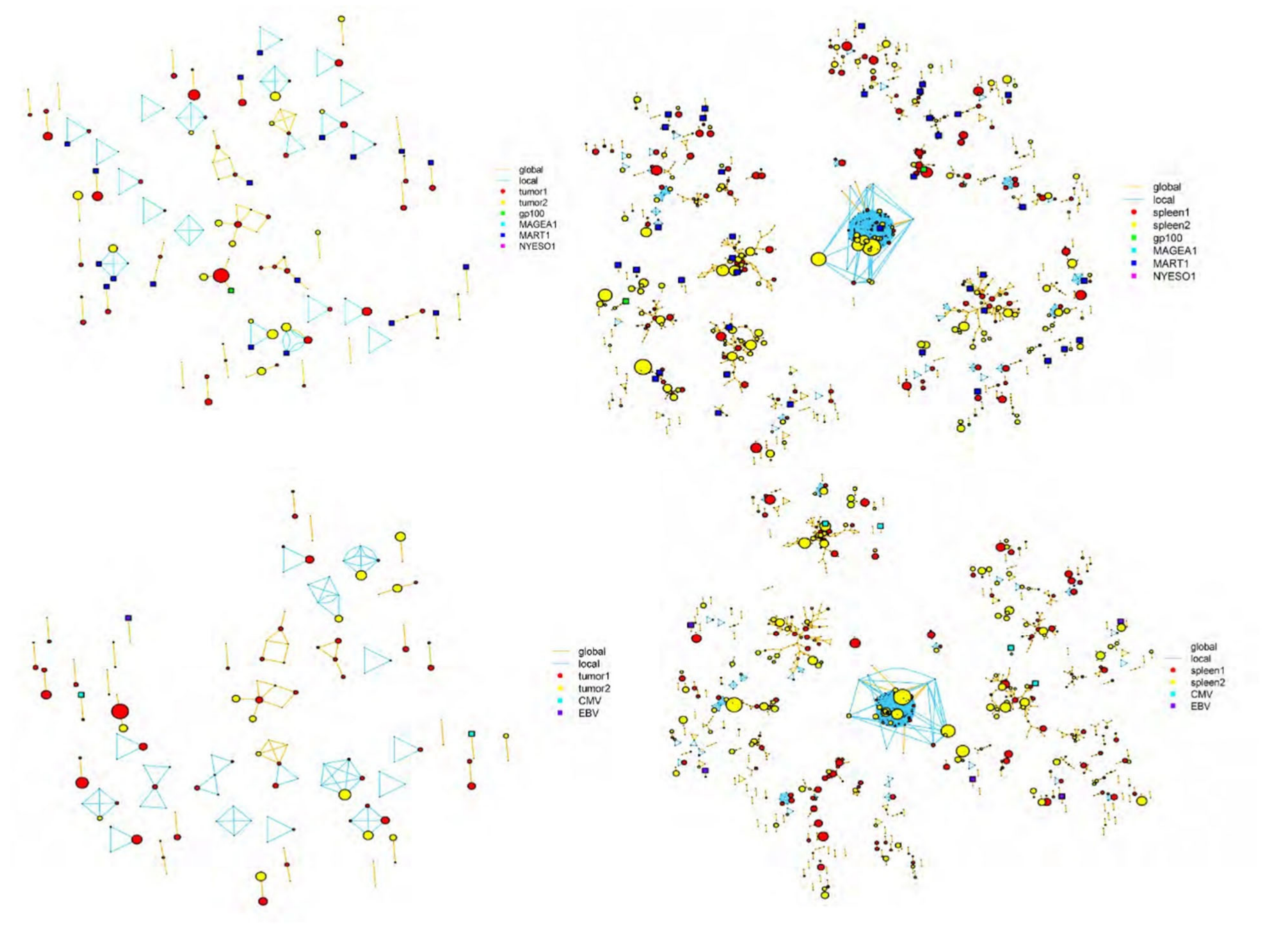

Supplementary Figure 7: Melanoma antigen-specific CDR3 (TCR) sequences are detected in TILs of Hu-mice. TCR sequences generated from TILs of two melanoma lesions (HLA-A2 matched to donor $CD34^+$ cells) or T cells from two spleens obtained from Hu-mice were clustered using the GLIPH algorithm (see method section) according to their local (**blue connections**) and global (**yellow connections**) similarity, which are likely to recognize a similar peptide/MHC complex. The TCRs are depicted as circles, the size indicating the abundance of the respective T-cell clonotype on a log scale. The origin, e.g. tumor 1 or 2 and spleen 1 or 2 is color coded. To predict the reactivity of the TCR clusters, virtual melanoma differentiation

antigens (MDA [MART-1 and gp100]), cancer testis antigens (CTA [MAGE A1 and NY-ESO-1]) were appended with fixed abundance values and depicted as squares (green, blue, cyan, and magenta, respectively). To determine specificity of CDR3 sequences virtual viral antigens (i.e., CMV and EBV) epitope/HLA-A2 reactive TCR were spiked in, which are depicted as color-coded squares **(cyan and violet**, respectively) with fixed abundance values.

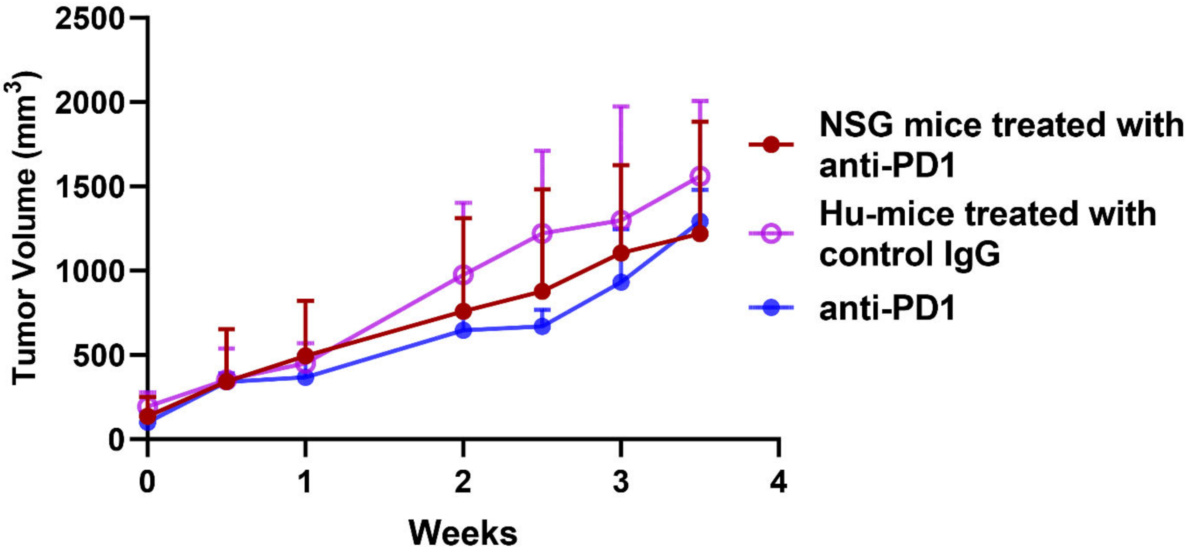

Supplementary Figure 8. Treatment with anti-PD-1 has no effect on aggressively growing melanoma tumor. In an established Hu-mice melanoma model (**see Fig. 2f**) anti-PD-1 treatment **(n=5)** was unable to restrict tumor growth of 451LU. Source data are provided as a Source Data file.

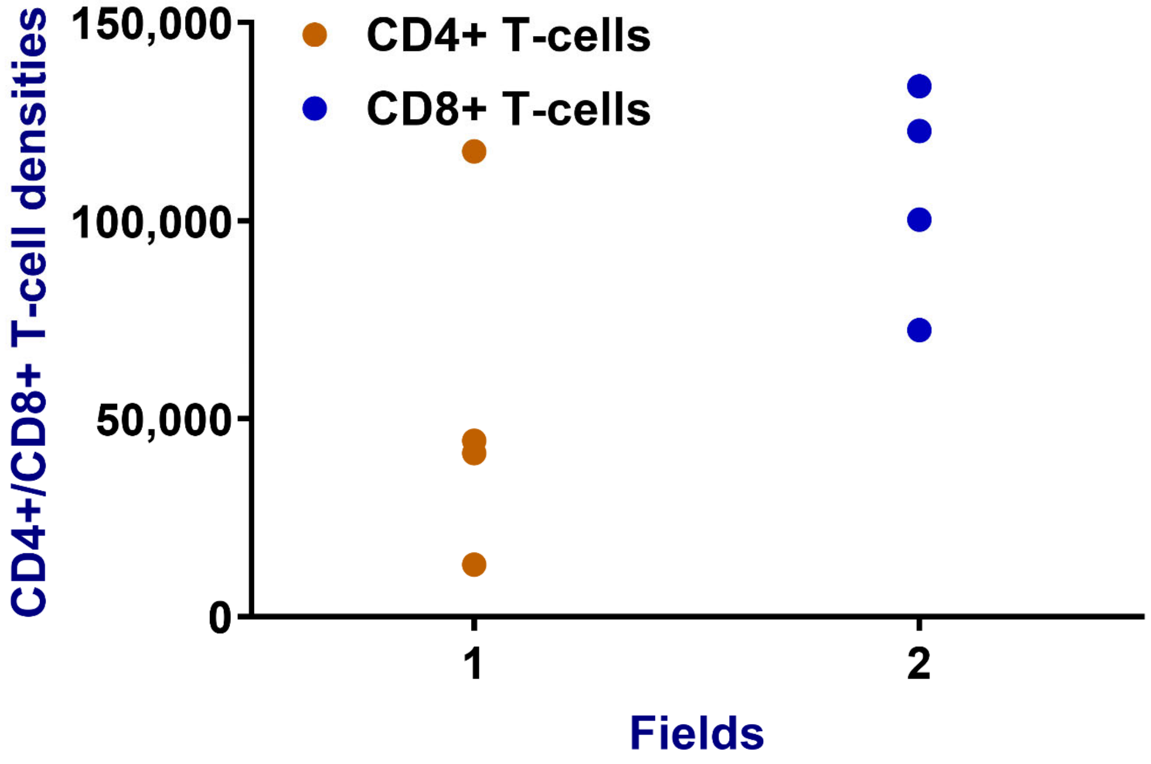

Supplementary Figure 9. Heterogeneous distribution of $CD4^+$ and $CD8^+$ T-cell distribution in tumors after anti-PD-1 therapy. Images of tumor sections were digitally quantified using NIS elements software and counts are from two different fields.

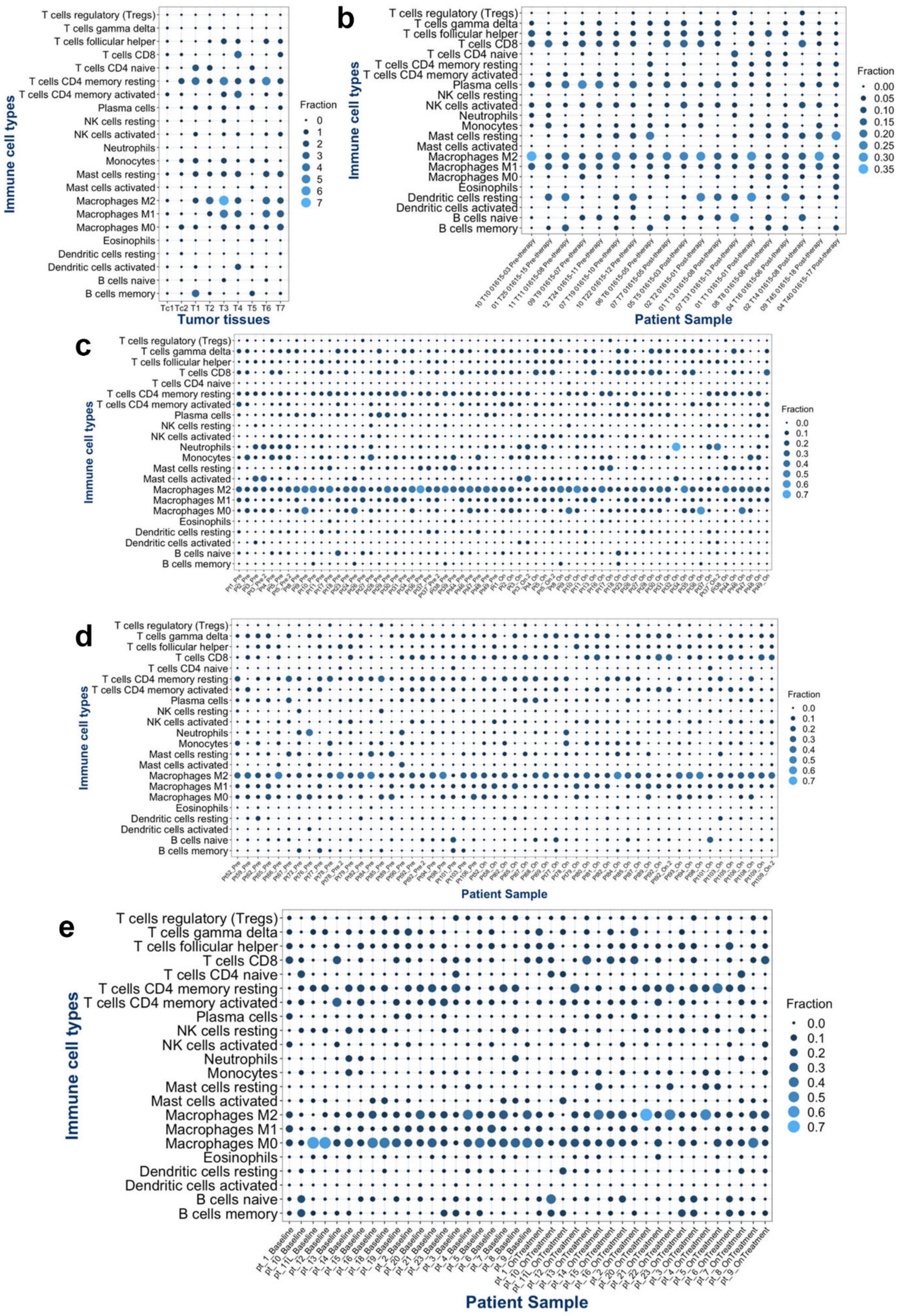
a
b
c
d
e
Immune cell types
T cells regulatory (Tregs)
T cells gamma delta
T cells follicular helper
T cells CD8
T cells CD4 naive
T cells CD4 memory resting
T cells CD4 memory activated
Plasma cells
NK cells resting
NK cells activated
Neutrophils
Monocytes
Mast cells resting
Mast cells activated
Macrophages M2
Macrophages M1
Macrophages M0
Eosinophils
Dendritic cells resting
Dendritic cells activated
B cells naive
B cells memory
Tc1 Tc2 T1 T2 T3 T4 T5 T6 T7
Tumor tissues
Fraction
0
1
2
3
4
5
6
7
Patient Sample
Fraction
0.00
0.05
0.10
0.15
0.20
0.25
0.30
0.35
Fraction
0.0
0.1
0.2
0.3
0.4
0.5
0.6
0.7

Supplementary Figure 10. Tumor-infiltrating immune cell types after immune-checkpoint therapy. CIBERSORT analysis of RNASeq from tumor tissues as in Figure 4a showed the presence of immune cell types including T- and B-cells, and monocytes/macrophages including mast cells. Some NK and DCs were also observed in the tumor tissues (**a**). **b-d**. Similarly, all 3 clinical trial datasets as in Figures 5b-d showed presence of other immune cell types. Size and the color of the closed circles indicates the abundance of each immune cell types in the tissues. Source data are provided as a Source Data file.

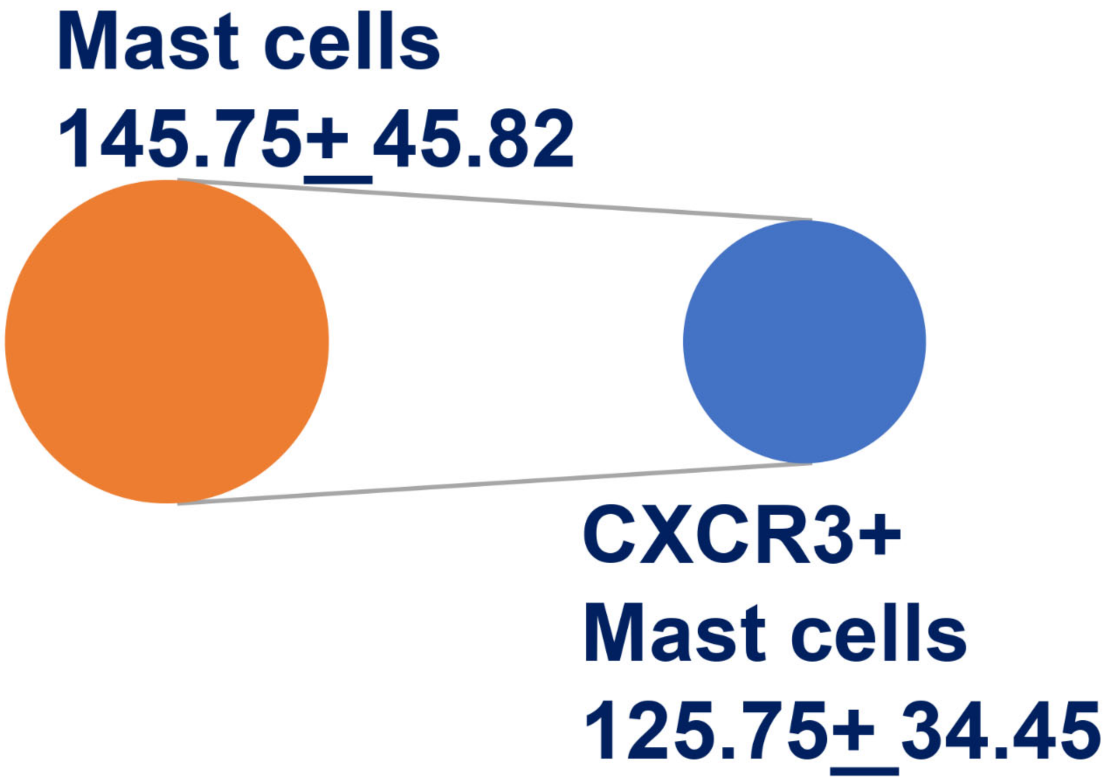

Supplementary Figure 11. Mast cells co-express CXCR3 after anti-PD-1 therapy. Increased levels of mast cells co-express CXCR3 after anti-PD-1 therapy, thus confirming their ability to migrate in response to increased CXCL-10 expression by tumor cells.

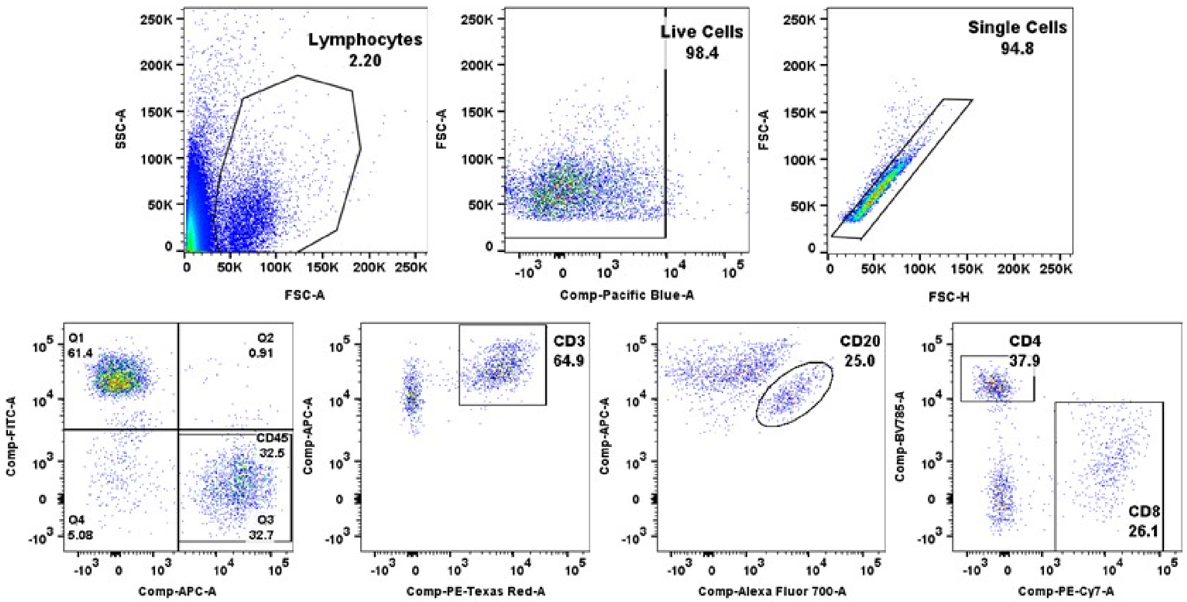

Supplementary Figure 12. Flow gating strategy. A representative example of flow strategy of Flow data in Fig.1b is described here. Immune cells labeled with antibody cocktail were analyzed by flow cytometry and acquired images are plotted using FlowJo software. Total lymphocytes are gated 1st (**top left panel)**, followed by gating for live cells **(top middle panel)**, and single cells (**top right panel**). From single cells, gating for mouse CD45^{+} cells (Y-axis) and human CD45^{+} cells (X-axis) are plotted (**bottom left panel)**. CD3^{+} (**2nd left panel**) , CD20^{+} (**3rd left panel**) and CD4^{+}/CD8^{+} (**right most panel**) cells are all derived from human CD45^{+} gated region.

Supplementary Table 1.

Antibody Reagents	Dilutions	Source	Catalog number
PE/Dazzle™ 594 mouse anti-human CD3; clone OKT3	1:25	BioLegend	317346
BV786 mouse anti-human CD4; clone SK3	1:25	BD Biosciences	563877
Rat anti-human CD4; clone A161A1	1:100	BioLegend	357402
PE-Cyanine7 mouse anti-human CD8a; clone SK1	1:25	ThermoFisher	25-0087-41
Mouse anti-human CD8; clone SK1	1:500	BioLegend	344702
APC-Cy™7 mouse anti-human CD11b; clone ICRF44	1:25	BD Biosciences	557754
APC mouse anti-human CD11b; clone ICRF44	1:25	BD Biosciences	550019
PE mouse anti-human CD14; clone 61-D3	1:25	ThermoFisher	12-0149-41
PerCP-Cy5.5 mouse anti-human CD15; clone W6D3	1:25	BioLegend	323020
Alexa Fluor 700 mouse anti-human CD20; clone 2H7	1:50	ThermoFisher	56-0209-42
Mouse anti-human CD20cy; clone L26	1:100	Dako	M0755
PE-Cy7 mouse anti-human CD33; P67.6	1:25	BD Biosciences	333946
APC mouse anti-human CD45; clone 2D1	1:50	ThermoFisher	17-9459-41
FITC rat anti-human CD45; clone 30-F11	1:25	ThermoFisher	11-0451-82
Mouse anti-human CD45; clone 2B11	1:300	Dako	M070101-2
BV605 mouse anti-human CD56; clone NCAM16.2	1:25	BD Biosciences	562780
Mouse anti-human CD68; clone Y1/82A	1:400	BioLegend	333801
Rabbit anti human CXCL10	1:100	ThermoFisher	PA5-79103
Mouse anti-human CXCR3; clone 49801	1:50	R&D Systems	MAB160-100
FITC mouse anti-human HLA-DR; clone G46-6	1:25	BD Biosciences	555811
Rabbit anti-human HLA class I	1:50	MyBiosource	MBS2524608
Mouse anti-human HMB45; clone HMB-45	1:50	Dako	M0634
Mouse anti-human mast cell tryptase	1:100	BioLegend	369402
FITC mouse anti-MHC Class I (H-2Kb); clone AF6-88.5.5.3	1:25	ThermoFisher	11-5958-82
Mouse anti-human TCR gamma/delta; clone B6	1:50	ThermoFisher	331402
Donkey anti-mouse IgG; Qdot625	1:500	ThermoFisher	Q22085
Alexa Fluor 488 goat anti-mouse IgG1	1:50	ThermoFisher	A-21121
Alexa Fluor 647 goat anti-mouse IgG2a	1:50	ThermoFisher	A-21241
Alexa Fluor 647 goat anti-mouse IgG	1:50	ThermoFisher	A-21236
Alexa Fluor 546 goat anti-rabbit IgG (H+L)	1:50	ThermoFisher	A-11035

Supplementary Table 2.

CyTOF Antibody Reagents	Dilutions	Mass	Source	Catalog number
Rat anti-human CD4; clone A161A1	1:100	156	BioLegend	357402
Mouse anti-human CD8; clone SK1	1:500	162	BioLegend	344702
Mouse anti-human CD45RO; clone UCHL1	1:300	173	BioLegend	304202
Mouse anti-human Granzyme B; clone 12F9B65	1:600	167	BioLegend	662801
Mouse anti-human FOXP3; clone 259D	1:25	155	BioLegend	320201
Mouse anti-human HLA class I; W6/32	1:50	141	BioLegend	311402
Mouse anti-human Nestin; clone 10C2	1:300	146	BioLegend	656802

Supplementary Table 3.

**Cytokine primers**	**Primer sequence**
IFNγ forward	TCTGCATCGTTTTGGGTTCT
IFNγ reverse	GCAGGCAGGACAACCAT-TACT
IL-2 forward	GAATGGAATTAATAATTACAAGAATC
IL-2 reverse	ATGTTGTTTCAGATCCCTTTAGTTCCAGA
GAPDH forward	GTCTCCTCTGACTTCAACAGCG
GAPDH reverse	ACCACCCTGTTGCTGTAGCCAA
