# Supplementary material for: Tumor-infiltrating mast cells are associated with resistance to anti-PD-1 therapy
Source: Nat Commun. 2021 Jan 12;12:346. doi: 10.1038/s41467-020-20600-7 (PMC7804257; doi:10.1038/s41467-020-20600-7)
Supplement: Supplementary file 2 — Reporting Summary [file 41467_2020_20600_MOESM2_ESM.pdf]

nature research

Corresponding author(s): Somasundaram et al

Last updated by author(s): Dec 8, 2020

Reporting Summary

Nature Research wishes to improve the reproducibility of the work that we publish. This form provides structure for consistency and transparency in reporting. For further information on Nature Research policies, see our Editorial Policies and the Editorial Policy Checklist.

Statistics

For all statistical analyses, confirm that the following items are present in the figure legend, table legend, main text, or Methods section.

n/a	Confirmed	
☐	☒	The exact sample size (*n*) for each experimental group/condition, given as a discrete number and unit of measurement
☐	☒	A statement on whether measurements were taken from distinct samples or whether the same sample was measured repeatedly
☐	☒	The statistical test(s) used AND whether they are one- or two-sided<br>*Only common tests should be described solely by name; describe more complex techniques in the Methods section.*
☒	☐	A description of all covariates tested
☒	☐	A description of any assumptions or corrections, such as tests of normality and adjustment for multiple comparisons
☐	☒	A full description of the statistical parameters including central tendency (e.g. means) or other basic estimates (e.g. regression coefficient) AND variation (e.g. standard deviation) or associated estimates of uncertainty (e.g. confidence intervals)
☐	☒	For null hypothesis testing, the test statistic (e.g. F, t, r) with confidence intervals, effect sizes, degrees of freedom and P value noted<br>*Give P values as exact values whenever suitable.*
☒	☐	For Bayesian analysis, information on the choice of priors and Markov chain Monte Carlo settings
☒	☐	For hierarchical and complex designs, identification of the appropriate level for tests and full reporting of outcomes
☒	☐	Estimates of effect sizes (e.g. Cohen's *d*, Pearson's *r*), indicating how they were calculated

Our web collection on statistics for biologists contains articles on many of the points above.

Software and code

Policy information about availability of computer code

Data collection	Dotblot Figure generation Source code provided.
Data analysis	GraphPad Prism 8, ImageJ Win64, MS EXCEL (Office 365 Pro Plus), NIS Elements AR 5.02, Flow Jo 10.7.1, RSEM v1.2.12, DESeq2, R v3.4.1, R Statistical package Applied 486 Biosystems 7500, v2.0, IMSEQ 497,

For manuscripts utilizing custom algorithms or software that are central to the research but not yet described in published literature, software must be made available to editors and reviewers. We strongly encourage code deposition in a community repository (e.g. GitHub). See the Nature Research guidelines for submitting code & software for further information.

Data

Policy information about availability of data

All manuscripts must include a data availability statement. This statement should provide the following information, where applicable:

- Accession codes, unique identifiers, or web links for publicly available datasets
- A list of figures that have associated raw data
- A description of any restrictions on data availability

Source Data is provided as Supplementary information and RNAseq dataset (GSE161353) is available in the public domain.

Field-specific reporting

Life sciences study design

All studies must disclose on these points even when the disclosure is negative.

Sample size	Sample size was determined using Power analysis (R Statistical package) based on the tumor size variations with help from Dr. Liu, our Biostatistician.
Data exclusions	In rare cases of unexpected animal deaths mid way through the experiment. Experiments involving drug therapy after tumor challenge there were some unexpected deaths.
Replication	Every experiment was repeated at least 2x, all attempts in replicating the experiments were successful.
Randomization	Humanized mice were randomized based on their human lymphocyte count and distributed into different experimental groups for the study. Only female mice were used in the study as CD34 take rate were better in female mice.
Blinding	Tumor measurements were taken by an independent researcher who was not involved in treatment process. Cages were coded for treatment purposes and researcher measuring the tumor is not aware of the codes.

Reporting for specific materials, systems and methods

We require information from authors about some types of materials, experimental systems and methods used in many studies. Here, indicate whether each material, system or method listed is relevant to your study. If you are not sure if a list item applies to your research, read the appropriate section before selecting a response.

Materials & experimental systems

n/a	Involved in the study	
☐	☒	Antibodies
☐	☒	Eukaryotic cell lines
☒	☐	Palaeontology and archaeology
☐	☒	Animals and other organisms
☐	☒	Human research participants
☒	☐	Clinical data
☒	☐	Dual use research of concern

Methods

n/a	Involved in the study	
☒	☐	ChIP-seq
☐	☐	Flow cytometry
☒	☐	MRI-based neuroimaging

Antibodies

Antibodies used

Details of the antibodies used in the study are provided in Supplementary Tables 1 and 2. Dilutions used, Catalog number and the manufacturer is provided in these Tables.

Validation

All antibodies used in flow cytometry and IHC staining were based on manufacturer's recommended concentrations. They were tested on normal tissue/blood or melanoma tumor samples before used in the experimental assays. IHC antibody concentrations were fine tuned based on tissue origin to provide minimum background staining.
Any new batches of antibodies were tested the same way before used in the experimental set up.

1. PE/Dazzle™ 594 mouse anti-human CD3; clone OKT3, dilution 1:25, BioLegend 317346 (https://www.biolegend.com/en-us/products/pe-dazzle-594-anti-human-cd3-antibody-11986).
2. BV786 mouse anti-human CD4; clone SK3, dilution 1:25, BD Biosciences 563877 (https://www.bdbiosciences.com/us/applications/research/t-cell-immunology/th-1-cells/surface-markers/human/pe-cy7-mouse-anti-human-cd4-sk3-also-known-as-leu3a/p/560909).
3. Rat anti-human CD4; clone A161A1, 1:100, BioLegend 357402 (https://www.biolegend.com/en-us/products/purified-anti-human-cd4-antibody-8431).
4. PE-Cyanine7 mouse anti-human CD8a; clone SK1, 1:25, ThermoFisher 25-0087-41 (https://www.thermofisher.com/antibody/product/CD8a-Antibody-clone-SK1-Monoclonal/25-0087-41).
5. Mouse anti-human CD8; clone SK1, 1:500, BioLegend 344702 (https://www.biolegend.com/en-us/products/purified-anti-human-cd8-antibody-6144).
6. APC-Cy™7 mouse anti-human CD11b; clone ICRF44, 1:25, BD Biosciences 557754 (https://www.bdbiosciences.com/us/solrSearch?text=557754).
7. APC mouse anti-human CD11b; clone ICRF44, 1:25, BD Biosciences 550019 (https://www.bdbiosciences.com/us/solrSearch?text=550019).
8. PE mouse anti-human CD14; clone 61-D3, 1:25, ThermoFisher 12-0149-41 (https://www.thermofisher.com/antibody/product/CD14-Antibody-clone-61D3-Monoclonal/12-0149-41).
9. PerCP-Cy5.5 mouse anti-human CD15; clone W6D3, 1:25, BioLegend 323020 (https://www.biolegend.com/en-us/products/percp-cyanine5-5-anti-human-cd15-ssea-1-antibody-4249).
10. Alexa Fluor 700 mouse anti-human CD20; clone 2H7, 1:50, ThermoFisher 56-0209-42 (https://www.thermofisher.com/antibody/product/CD20-Antibody-clone-2H7-Monoclonal/56-0209-42).
11. Mouse anti-human CD20cy; clone L26, 1:100, Dako M0755 (https://www.agilent.com/en/product/immunohistochemistry/antibodies-controls/primary-antibodies/cd20cy-(dako-omnis)-76218).

12. PE-Cy7 mouse anti-human CD33; P67.6, 1:25, BD Biosciences 333946 (https://www.bdbiosciences.com/us/solrSearch text=333946).
13. APC mouse anti-human CD45; clone 2D1, 1:50, ThermoFisher 17-9459-41 (https://www.thermofisher.com/antibody/product/CD45-Antibody-clone-2D1-Monoclonal/17-9459-41).
14. FITC rat anti-human CD45; clone 30-F11, 1:25, ThermoFisher 11-0451-82 (https://www.thermofisher.com/antibody/product/CD45-Antibody-clone-30-F11-Monoclonal/11-0451-82).
15. Mouse anti-human CD45; clone 2B11, 1:300, Dako M070101-2 (https://www.agilent.com/en/product/immunohistochemistry/antibodies-controls/primary-antibodies/cd45-leucocyte-common-antigen-(dako-omnis)-76270).
16. BV605 mouse anti-human CD56; clone NCAM16.2 ,1:25, BD Biosciences 562780 (https://www.bdbiosciences.com/us/applications/research/stem-cell-research/hematopoietic-stem-cell-markers/human/negative-markers/bv605-mouse-anti-human-cd56-ncam162-also-known-as-ncam-16/p/562780).
17. Mouse anti-human CD68; clone Y1/82A, 1:400, BioLegend 333801 (https://www.biolegend.com/en-us/products/purified-anti-human-cd68-antibody-4835).
18. Rabbit anti human CXCL10, 1:100, ThermoFisher PA5-79103 (https://www.thermofisher.com/antibody/product/CXCL10-IP-10-Antibody-Polyclonal/PA5-79103).
19. Mouse anti-human CXCR3; clone 49801, 1:50, R&D Systems MAB160-100 (https://www.rndsystems.com/products/human-cxcr3-antibody-49801_mab160).
20. FITC mouse anti-human HLA-DR; clone G46-6, 1:25, BD Biosciences 555811 (https://www.bdbiosciences.com/us/applications/research/stem-cell-research/mesenchymal-stem-cell-markers-bone-marrow/human/negative-markers/fitc-mouse-anti-human-hla-dr-g46-6/p/555811).
21. Rabbit anti-human HLA class I, 1:50, MyBiosource MBS2524608 (https://www.mybiosource.com/polyclonal-human-antibody/hla-class-i/2524608).
22. Mouse anti-human HMB45; clone HMB-45, 1:50, Dako M0634 (https://www.agilent.com/cs/library/packageinsert/public/303299EFG_03.pdf).
23. Mouse anti-human mast cell tryptase, 1:100, BioLegend 369402 (https://www.biolegend.com/en-us/products/purified-anti-human-mast-cell-tryptase-antibody-12681).
24. FITC mouse anti-MHC Class I (H-2Kb); clone AF6-88.5.5.3, 1:25, ThermoFisher 11-5958-82 (https://www.thermofisher.com/antibody/product/MHC-Class-I-H-2Kb-Antibody-clone-AF6-88-5-5-3-Monoclonal/11-5958-82).
25. Mouse anti-human TCR gamma/delta; clone B6, 1:50, ThermoFisher 331402 (https://www.thermofisher.com/antibody/product/Rabbit-anti-Goat-IgG-H-L-Secondary-Antibody-Polyclonal/31402).
26. Donkey anti-mouse IgG; Qdot625, 1:500, ThermoFisher Q22085 (https://www.thermofisher.com/antibody/product/Donkey-anti-Mouse-IgG-H-L-Secondary-Antibody-Polyclonal/Q22085).
27. Alexa Fluor 488 goat anti-mouse IgG1, 1:50, ThermoFisher A-21121 (https://www.thermofisher.com/antibody/product/Goat-anti-Mouse-IgG1-Cross-Adsorbed-Secondary-Antibody-Polyclonal/A-21121).
28. Alexa Fluor 647 goat anti-mouse IgG2a, 1:50, ThermoFisher A-21241 (https://www.thermofisher.com/antibody/product/Goat-anti-Mouse-IgG2a-Cross-Adsorbed-Secondary-Antibody-Polyclonal/A-21241).
29. Alexa Fluor 647 goat anti-mouse IgG ,1:50, ThermoFisher A-21236 (https://www.thermofisher.com/antibody/product/Goat-anti-Mouse-IgG-H-L-Highly-Cross-Adsorbed-Secondary-Antibody-Polyclonal/A-212360).
30. Alexa Fluor 546 goat anti-rabbit IgG (H+L), 1:50, ThermoFisher A-11035 (https://www.thermofisher.com/antibody/product/Goat-anti-Rabbit-IgG-H-L-Highly-Cross-Adsorbed-Secondary-Antibody-Polyclonal/A-11035).
31. Mouse anti-human CD45RO; clone UCHL1, 1:300 , BioLegend 304202 (https://www.biolegend.com/en-us/products/purified-anti-human-cd45ro-antibody-860).
32. Mouse anti-human Granzyme B; clone 12F9B65, 1:600 , BioLegend 662801 (https://www.biolegend.com/en-us/products/purified-anti-granzyme-b-antibody-10419).
33. Mouse anti-human FOXP3; clone 259D, 1:25, BioLegend 320201 (https://www.biolegend.com/en-us/products/purified-anti-human-foxp3-antibody-2903).
34. Mouse anti-human Nestin; clone 10C2, 1:300, BioLegend 656802 (https://www.biolegend.com/en-us/products/purified-anti-nestin-antibody-8836).

Eukaryotic cell lines

Policy information about cell lines

Cell line source(s)	Tumor cell lines were originally obtained from ATCC (A375) or previously established at Wistar (WM3629, 451LU).
Authentication	All cell lines were tested for short tandem repeat profile (DNA identity) before use in the experiments.
Mycoplasma contamination	All cell lines were tested for mycoplasma negativity before use in the experiments.
Commonly misidentified lines (See ICLAC register)	none used.

Animals and other organisms

Policy information about studies involving animals; ARRIVE guidelines recommended for reporting animal research

Laboratory animals	Immunodeficient NSG female mice (6-8 weeks) that are bred at Wistar were used for the study. Humanized mice were generated in house using NSG mice. All animal protocols were approved by IACUC. Details on housing conditions are provided in the Method section of the manuscript.
Wild animals	No wild animals were used.

Field-collected samples	No field collected samples were used in the study
Ethics oversight	All animal protocols are approved by Wistar IACUC.

Note that full information on the approval of the study protocol must also be provided in the manuscript.

Human research participants

Policy information about studies involving human research participants

Population characteristics	No human subjects were recruited specifically for this study. Melanoma tumor were obtained from Caucasian population that was from a previously published study. Fetal tissue were procured from a non-profit source (Advanced Bioscience Resources, Alameda, CA. Demographics of this population is not known.
Recruitment	NA
Ethics oversight	NA

Note that full information on the approval of the study protocol must also be provided in the manuscript.

Flow Cytometry

Plots

Confirm that:

- [x] The axis labels state the marker and fluorochrome used (e.g. CD4-FITC).
- [x] The axis scales are clearly visible. Include numbers along axes only for bottom left plot of group (a 'group' is an analysis of identical markers).
- [x] All plots are contour plots with outliers or pseudocolor plots.
- [x] A numerical value for number of cells or percentage (with statistics) is provided.

Methodology

Sample preparation	Sample preparation was performed according to our previous publication Somasundaram et al 2017. In brief, 100 ul Hu-mouse<br>blood was subjected to red blood cell lysis using ACK buffer, followed by incubation with human fluorochrome conjugated antibodies. Excess antibody was washed off, cells counter stained with DAPI and ready to run on the flow cytometer.
Instrument	LSR II Analyzer BD BioSciences.
Software	Raw flow data were analyzed by using FLOWJO 10.5.2.
Cell population abundance	Human CD45+ population was gated out from mouse CD45+ cells.
Gating strategy	Total lymphocyte gating was performed using FSC/SSC, live and single cells were selected and then the cells were gated for mouse and human CD45+ cells.

- [x] Tick this box to confirm that a figure exemplifying the gating strategy is provided in the Supplementary Information.
